# An mHealth Intervention With Financial Incentives to Promote Smoking Cessation and Physical Activity Among Black Adults: Protocol for a Feasibility Randomized Controlled Trial

**DOI:** 10.2196/69771

**Published:** 2025-01-31

**Authors:** Adam Alexander, Michael Businelle, Marshall Cheney, Amy Cohn, Lorna McNeill, Kevin Short, Summer Frank-Pearce, David Bradley, Kimberly Estrada, Iván Flores, Jack Fronheiser, Darla Kendzor

**Affiliations:** 1 University of Oklahoma Health Sciences Center Oklahoma City, OK United States; 2 University of Oklahoma Norman, OK United States; 3 The University of Texas MD Anderson Cancer Center Houston, TX United States

**Keywords:** African American, Black, mobile health, mHealth, smartphone app, smoking cessation, physical activity, mobile phone

## Abstract

**Background:**

Black adults in the United States experience disproportionately high rates of tobacco- and obesity-related diseases, driven in part by disparities in smoking cessation and physical activity. Smartphone-based interventions with financial incentives offer a scalable solution to address these health disparities.

**Objective:**

This study aims to assess the feasibility and preliminary efficacy of a mobile health intervention that provides financial incentives for smoking cessation and physical activity among Black adults.

**Methods:**

A total of 60 Black adults who smoke (≥5 cigarettes/d) and are insufficiently physically active (engaging in <150 min of weekly moderate-intensity physical activity) will be randomly assigned to either HealthyCells intervention (incentives for smoking abstinence only) or HealthyCells+ intervention (incentives for both smoking abstinence and daily step counts). Participants will use study-provided smartphones, smartwatches, and carbon monoxide monitors for 9 weeks (1 wk prequit date through 8 wk postquit date). Feasibility will be evaluated based on recruitment rates, retention, and engagement. The primary outcomes include carbon monoxide–verified, 7-day smoking abstinence at 8 weeks postquit date and changes in average daily step count. Feasibility benchmarks include a recruitment rate of ≥5 participants per month, a retention rate of ≥75%, and a smoking abstinence rate of ≥20% at 8 weeks postquit date. Expected increases in physical activity include a net gain of 500 to 1500 steps per day compared to baseline.

**Results:**

Recruitment is expected to begin in February 2025 and conclude by September 2025, with data analysis completed by October 2025.

**Conclusions:**

This study will evaluate the feasibility of a culturally tailored mobile health intervention combining financial incentives for smoking cessation and physical activity promotion. Findings will inform the design of larger-scale trials to address health disparities through scalable, technology-based approaches.

**Trial Registration:**

ClinicalTrials.gov NCT05188287; https://clinicaltrials.gov/ct2/show/NCT05188287

**International Registered Report Identifier (IRRID):**

PRR1-10.2196/69771

## Introduction

### Background

In the United States, tobacco- and obesity-related diseases are among the top 10 leading causes of death in the Black and African American (henceforth Black) population, and these diseases are more common and deadlier among Black adults compared with the overall US population [1-3]. For example, while smoking prevalence is similar among Black and White adults, Black adults are less likely to quit smoking than other racial and ethnic groups [4-6], and rates of lung cancer incidence and mortality are higher among Black male individuals than White male individuals [3]. Likewise, the Black population has high rates of obesity in the United States [7]. Physical inactivity (ie, no physical activity outside of work during the past month), a leading cause of obesity [8,9], is higher among Black adults compared with most other racial groups [8,9]. High rates of smoking (14.2%) [10] and insufficient physical activity (30%) [11] among Black adults are important modifiable health risk factors for cancer and chronic illness [12]. Interventions that simultaneously address these behaviors may promote holistic lifestyle changes, provide significant health benefits, and increase health equity within the Black population [13,14].

Multiple health behavior change (MHBC) interventions are defined as those that promote 2 or more health behaviors and thus have a more comprehensive focus on lifestyle health promotion [15,16]. Smoking and low physical activity are strong candidates for these interventions because people who smoke generally report less physical activity than those who do not smoke [17], and individuals may benefit from replacing smoking with physical activity during a quit attempt [18]. Importantly, engaging in physical activity may increase self-efficacy for quitting and alleviate cigarette cravings during withdrawal [19-22]. Physical activity also improves mood and reduces stress, which are common triggers for smoking relapse [23]. However, numerous studies have shown mixed results regarding the physical activity’s effectiveness for smoking cessation [24], in part because adherence to physical activity varies significantly across individuals [23,25,26], with stronger adherence among adults with existing physical activity habits and strong social support, and the type of activity (eg, low-intensity activity vs high-intensity activity) [27].

Low-intensity activities, such as walking, may be more practical and palatable for people trying to quit smoking while still offering benefits for smoking cessation, such as reduced cravings and stress relief [13,19,28]. Low-intensity activities may also promote greater adherence to physical activity because they are accessible, comfortable, and less likely to cause fatigue or injury than high-intensity workouts [29]. Furthermore, low-intensity activities can be included in daily routines, such as walking to a bus stop or church [30]. The inclusivity and ease of low-intensity physical activity may encourage long-term engagement and consistency. Thus, combining low-intensity physical activity with other cessation strategies may synergistically increase the likelihood of smoking cessation and have a greater impact on overall health.

Contingency management (CM) is a simple, powerful, and widely used behavioral strategy where tangible rewards are offered for meeting a prespecified behavior change goal [31-33]. CM is efficacious for improving smoking cessation and physical activity [34-38], but common concerns about CM are intervention costs and maintenance of long-term behavior change once incentives cease [39]. Yet, recent research on smoking and physical activity has shown that CM interventions are cost-effective, and even small financial incentives can lead to long-term changes in smoking and physical activity after incentives are withdrawn [34-38]. Thus, an MHBC intervention that incentivizes behavior change could help Black adults initiate and sustain smoking cessation and adequate physical activity in conjunction with other recommended tobacco treatment components (ie, counseling and pharmacotherapy) [40].

Despite the rapid growth of mobile health (mHealth) technologies, such as smartphone apps, that offer new opportunities to reach and treat at-risk populations, Black adults remain underrepresented in mHealth research [41]. As of 2024, 84% of the Black population owned a smartphone [42]; therefore, app-based interventions have the potential to increase the reach and use of effective treatments for smoking and physical activity within this population. Furthermore, apps combined with sensors and wearables can be powerful tools for self-regulation [42], which is a core component of behavior change [43], and can provide frequent feedback and engagement opportunities. Initial evidence suggests that app-based interventions may be a useful supplement to more intensive behavioral or pharmacological treatments and may even show comparable outcomes to traditional face-to-face interventions in some cases [44-47]. Furthermore, smartphone apps can be used to characterize the behaviors, needs, and outcomes of traditionally understudied and underserved groups.

Research indicates that interventions incorporating culturally relevant content resonate with Black adults, enhancing their motivation and commitment to behavior change [48-53]. Such content has increasingly been incorporated into mHealth interventions targeting smoking cessation and physical activity among Black adults [54-57]. Drawing from publicly available resources such as *Pathways to Freedom—*a short documentary highlighting the historical and cultural ties between the Black community and tobacco use and incorporating health education and culturally relevant messages to motivate Black adults to quit smoking [48,49]—mHealth apps can provide educational content on the historical and social influences of smoking within the Black community. mHealth apps can also provide motivational messages and stress-management techniques grounded in culturally relevant themes, such as prayer and seeking support from family and friends. Furthermore, app-based interventions can deliver education about the purpose and benefits of nicotine replacement therapy (NRT) and debunk myths and misconceptions about NRT that are present in this population [58-60]. Apps can also make it easier for Black adults to create activity goals that reflect their current life circumstances and environment [61,62], and these goals can be adjusted dynamically based on daily performance and feedback, ensuring that goals remain challenging yet attainable. This content can be presented alongside success stories from notable and relatable Black figures to provide additional motivational support [63-65]. Altogether, when delivered with evidence-based treatment for smoking cessation and physical activity, culturally relevant content may reinforce the quit journey and help address common challenges that Black adults face when quitting smoking and engaging in physical activity.

### Objectives

The purpose of this project is to explore the feasibility of an app-based MHBC intervention (HealthyCells) for Black adults who smoke and are insufficiently physically active. In addition to evidence-based tobacco cessation treatment [66], HealthyCells will incentivize daily smoking abstinence, while an enhanced version of the intervention, HealthyCells+, will also include incentives for meeting physical activity goals [36,67]. The inclusion of 2 active study conditions allows for a direct comparison to evaluate the added benefit of financial incentives for physical activity. The central hypothesis is that participants in the HealthyCells+ group will demonstrate greater increases in daily physical activity (measured via step counts) and improved smoking abstinence rates compared to those receiving smoking cessation incentives alone (HealthyCells). HealthyCells will also include an interactive educational e-book experience inspired by the documentary *Pathways to Freedom* [48,49], designed to guide individuals during their quit attempt and provide opportunities to set personalized activity goals, which can be dynamically adjusted daily to reflect current readiness. The primary aim will be to demonstrate intervention feasibility, including recruitment, retention, and adherence metrics, along with rates of carbon monoxide (CO)–verified smoking abstinence and prequit and postquit changes in physical activity.

## Methods

### Overview

Black adults who smoke and are insufficiently physically active (ie, not meeting physical activity guidelines) [68] will be randomly assigned to 1 of the 2 versions of an MHBC intervention that targets smoking cessation and insufficient physical activity: (1) HealthyCells (incentives for smoking abstinence only) or (2) HealthyCells+ (incentives for both smoking abstinence and physical activity). Both groups will receive a comprehensive tobacco treatment intervention, including 8 weeks of NRT, an initial counseling session to create a quit plan, and 5 weekly counseling sessions (6 sessions in total). Likewise, all participants will receive a study-provided smartphone and intervention app, a Samsung smartwatch to measure daily steps, and a Bedfont iCOquit Smokerlyzer to verify smoking status. Participants will be asked to use their assigned app and equipment for 9 weeks (1 wk prequit date through the eighth wk postquit date).

### Ethical Considerations

This study was approved by the institutional review board of the University of Oklahoma Health Sciences Center (protocol #14094), and the study was registered at ClinicalTrials.gov (NCT05188287). All procedures involving human participants will adhere to the Declaration of Helsinki and relevant institutional guidelines. Written informed consent will be obtained digitally from all participants before enrollment, and they will be informed of their right to withdraw from the study at any time without penalty. To ensure privacy and confidentiality, participant data will be deidentified and stored on a secure, password-protected server. All breath sample photos and step count uploads will be encrypted, with access restricted to authorized research personnel. Following a structured and transparent payment schedule, participants will receive compensation based on their engagement with study activities, including breath sample submissions, step count verifications, surveys, and interviews (refer to the Study Compensation section). To protect participant privacy, all devices used for data collection, including Samsung smartphones and smartwatches, will be reset to factory settings after study participation has ended for each participant. Any adverse events or deviations from the protocol will be promptly reported to the institutional review board in compliance with ethical oversight requirements.

### Theoretical Framework

**Figure 1:**
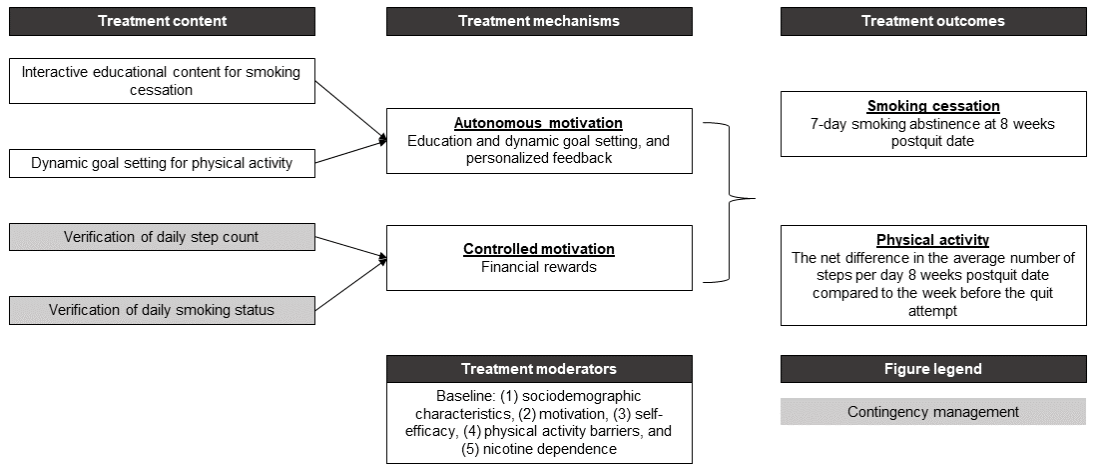
The theoretical framework of HealthyCells app, a multiple health behavior change intervention for smoking cessation and physical activity.

As shown in Figure 1, this intervention will be guided by self-determination theory principles [69-72]. Self-determination theory posits that two forms of motivation are involved in behavior change: (1) autonomous motivation and (2) controlled motivation [72]. Autonomous motivation consists of engaging in behavior out of curiosity, interest, a sense of challenge, or for its inherent enjoyment. Conversely, controlled motivation involves engaging in a behavior because of external rewards or punishment avoidance [72]. Autonomous motivation is associated with sustained behavior change, whereas controlled motivation is related to temporary change [73]. CM interventions provide financial rewards for immediate behavior change, primarily targeting controlled motivation [74].

Incentives can be paired with resources and activities that cultivate autonomous motivation to sustain long-term behavior change [75]. The research team created an interactive educational e-book experience inspired by *Pathways to Freedom* [48,49] intervention that focuses on several key topics highlighted in Textbox 1, such as the harms of smoking, tobacco marketing, NRT education, and stress management, and includes narratives from several prominent Black individuals who have struggled with smoking, such as the former US President Barack Obama [76]. The research team also developed a personalized activity goal algorithm based on findings from mHealth physical activity interventions [54,77] and qualitative interviews with Black adults [61,78].

**Overview of topics covered in the HealthyCells, Healthy Habit e-book.**

**Chapters and interactive educational content**

- Chapter 1: discusses health and economic harms caused by smoking and the benefits after quitting
- Chapter 2: covers the hidden history of Black adults and the tobacco industry
- Chapter 3: describes how habits are created and why smoking is one of the deadliest habits to break
- Chapter 4: provides an overview of how nicotine reinforces the smoking habit
- Chapter 5: reviews the purpose and benefits of nicotine replacement therapy

### Inclusion and Exclusion Criteria

Inclusion criteria for the participants will be as follows: (1) resident of Oklahoma (verified via driver’s license or ID); (2) aged ≥18 years; (3) self-identify as *Black* or *African American*, either alone or in combination with other races or Hispanic ethnicity; (4) smoke ≥5 cigarettes or cigarette equivalents per day (ie, cigars and cigarillos); (5) currently (per self-report) engage in <150 minutes of moderate-intensity aerobic physical activity or <75 minutes of vigorous-intensity aerobic physical activity per week [68]; (6) willing to use NRT, a smartwatch, or the study app and able to attend smoking cessation counseling; (7) willing to quit smoking within the next 3 weeks; and (8) exhaled CO level of >6 ppm at app activation.

Exclusion criteria for the participants will be as follows: (1) unwilling to complete the screening survey, (2) unable to understand or speak English, (3) not a US citizen or permanent resident (due to university taxation policies), (4) unwilling to provide social security number, US residency status, or university employee status (to adhere to university reporting requirements), (5) unwilling to abstain from smoking marijuana or cannabis during their quit attempt, (6) unwilling to provide a picture of a tobacco product (to provide initial evidence of current smoking), (7) medical condition (per self-report) that is a potential contraindication for NRT use (eg, uncontrolled hypertension, heart disease, or recent heart attack), or (8) has had a severe allergic reaction or side effect from the use of NRT.

### Recruitment

**Figure 2:**
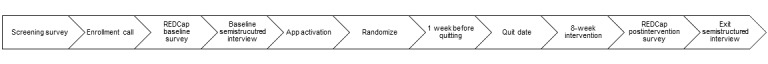
Participant flow. REDCap: Research Electronic Data Capture.

Participants will be remotely recruited and enrolled. No in-person visits are required (Figure 2). Eligible individuals will be identified through the Tobacco Treatment Research Program [79], which offers free tobacco cessation support to adult Oklahomans and assists with study recruitment [38,80-82]. Prospective participants will also be contacted by SMS text messages, email, and phone, and recruitment flyers will be posted in community locations (eg, barbershops and churches) and on social media through a geotargeted campaign focused on Black adults who smoke. Those interested can access a brief web-based eligibility screener via a QR code or direct link.

### Study Enrollment

Study staff will contact via phone adults who meet preliminary eligibility to confirm eligibility and discuss study participation. To prevent fraudulent enrollments [83,84], participants will be asked to provide a photo of their driver’s license or ID (or other proof of address) and an image of a personal tobacco product to provide initial evidence of smoking status. Eligible and willing participants will be informed about the study and asked to acknowledge their consent with a digital signature. Following enrollment, participants will complete a baseline survey via REDCap (Research Electronic Data Capture; Vanderbilt University) [85] (refer to Multimedia Appendix 1 for a complete list of study measures), and their first semistructured phone interview will be scheduled to discuss their openness to using smartphone apps and to identify potential barriers to mHealth research participation (Table 1). After the interview, participants will receive a welcome packet with study instructions, copies of informed consent, and other signed study documentation, a reloadable Greenphire ClinCard [86], intervention equipment (smartphone, smartwatch, and Bedfont iCOquit Smokerlyzer), and NRT by mail.

**Table 1:** Overview of qualitative domains covered in each interview.

Domain	Baseline interview	Exit interview
Participation in mHealth^a^ research	✓	
Understanding smartphone use and habits	✓	
History of using health apps and Bluetooth-enabled devices	✓	
History of using apps that promote smoking cessation and physical activity	✓	
Understanding preferences for smartphone apps that promote health behavior change within the Black population	✓	✓
History of quitting smoking and the methods used to quit smoking before study participation		✓
Problems with study equipment and smartphone app		✓
Overall impressions of app features		✓
Experiences using smartwatch during study participation		✓
Experiences using NRT^b^ during study participation		✓

^a^mHealth: mobile health.

^b^NRT: nicotine replacement therapy.

Upon confirming receipt of the study phone and materials, participants will complete an activation assessment in the study app, independently or with study staff. This will assess their ability to use app features, including viewing images, hearing sounds, reading text, and using the Bedfont iCOquit Smokerlyzer. Participants with an expired breath CO <6 ppm or who cannot use the Bedfont iCOquit Smokerlyzer will be withdrawn before randomization and asked to return the study phone and smartwatch. Participants can keep the NRT and Bedfont iCOquit Smokerlyzer to support their quit attempt and will be transferred to the Tobacco Treatment Research Program for cessation services. Participants who pass the activation assessment will be randomly assigned to the HealthyCells or HealthyCells+ intervention.

### Study Randomization

Participants will be stratified according to self-reported sedentary time at baseline (high: >8 h/d, medium: 4-8 h/d, and low: <4 h/d) assessed via the International Physical Activity Questionnaire [87]. Participants (n=60) will be randomized 1:1 in REDCap [85], and research staff will text each new participant a unique code (HealthyCells: 0101 and HealthyCells+: 0202) to access assigned app content.

### Standard Care

All participants will receive standard intensive tobacco treatment [66], beginning with a phone counseling session 1 week before the scheduled quit date and continuing weekly through the fourth week after the quit date. Counseling sessions will cover topics, including creating a quit plan, the health impact of tobacco, stress management, adopting lifestyle changes, developing coping strategies, and relapse prevention. In addition, participants will receive 8 weeks of combination NRT (nicotine patches+gum or lozenges) based on package-recommended dosing tailored to baseline smoking and nicotine dependence levels [88]. The first 4-week supply will be mailed, and participants can request the remaining supply through the study app as needed.

### MHBC Intervention: App Development and Pilot Testing

#### Overview

The HealthyCells app was developed using the Insight mHealth Platform [47]. All app content, including breath sample submissions, step count tracking, and interactive educational material, was presented to the African American Cancer Research Community Advisory Board at the Stephenson Cancer Center in November 2024 to solicit community feedback. This meeting was digitally recorded, and the study team reviewed transcripts to identify strengths, weaknesses, and gaps where the app content could be refined. Additional systematic usability testing will be conducted in this feasibility trial to evaluate app engagement, functionality, and acceptability among participants. Findings from this study will inform further refinements to the app in preparation for future large-scale trials to maximize its effectiveness and user experience.

#### MHBC Intervention: HealthyCells

In addition to all components of standard care described earlier, HealthyCells participants will be able to earn daily financial incentives for CO-verified smoking abstinence each day. Smoking status and identity will be assessed remotely via (1) self-reported daily smoking status, (2) CO breath sample submissions (ie, via the Bedfont iCOquit Smokerlyzer), and (3) Microsoft Azure facial recognition software; each of these assessment types has been successfully integrated into the Insight mHealth Platform [89,90]. Participants will be asked to upload a photo of themselves during the app activation phase, which will be stored as the reference photo for future identity verification. Participants will be instructed to keep their appearance consistent with the baseline photo during breath sample submissions (eg, removing or staying consistent with glasses, hats, and ponytails). During breath sample submissions, 2 photographs will be taken randomly during the exhalation phase to verify identity. Photographs will be encrypted and stored on the study server for later viewing by study staff as needed. A facial recognition mismatch will pause the delivery of incentives and will be flagged for internal review by study staff. Staff will click “thumbs up” or “thumbs down” for an identity match or mismatch, and incentives will be earned on schedule or unearned with incentive levels reset to the starting level.

**Figure 3:**
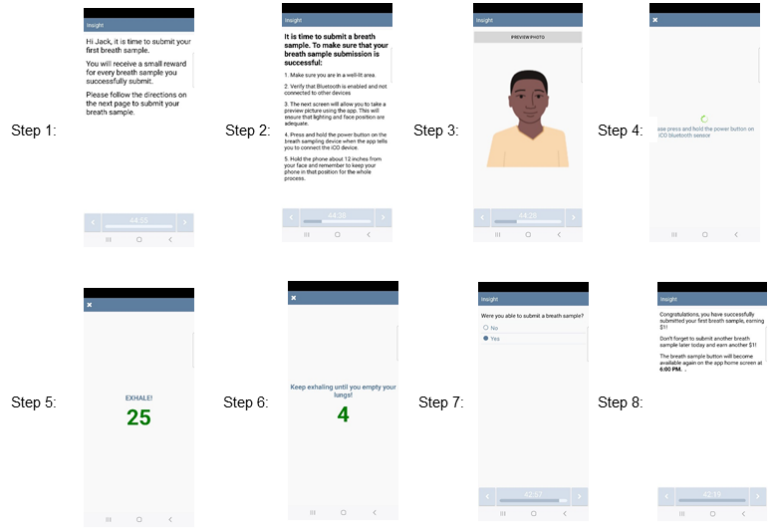
An example of a breath sample submission to verify smoking status.

To earn incentives for smoking abstinence, participants will be instructed to submit 2 breath samples each day (Figure 3). These participant-initiated assessments will appear on the home menu screen, with the first assessment appearing at the beginning of each day (based on participants’ self-reported waking hours) and the second assessment appearing 8 hours after the initial evaluation is completed. Participants will earn US $1 for each breath sample submission regardless of current smoking status. The incentive schedule used in this study is an adaptation of a low-cost incentive schedule the investigators have used in their previous and ongoing work with adults who are socioeconomically disadvantaged [38,89-91]. Participants who demonstrate abstinence (ie, self-reported abstinence that day, CO ≤6 ppm, and identity verification) on both breath sample submissions on their scheduled quit date will receive US $20 to reinforce the initiation of the quit attempt strongly. After that, abstinent participants will be rewarded US $4 per day, increasing by US $0.50 per week until US $5.50 per day is reached during the fourth week postquit date. Participants who are nonabstinent (or who do not provide a sample) will not earn an incentive that day, though they may begin earning incentives for abstinence again the next day. However, the amount will reset to the starting level of US $4 per abstinent day. Participants will earn US $7 per abstinent day during the eighth week postquit date. To gain the maximum incentives for smoking cessation (US $325), participants must report daily smoking abstinence and submit 2 breath samples each day (CO ≤6 ppm) with identity verification during all eligible assessment weeks (ie, wk 1-4 and the eighth wk postquit date).

HealthyCells intervention participants will receive a Samsung smartwatch to track their activity and a daily SMS text message about the benefits of increasing physical activity via the app (refer to the Daily Smoking Cessation Progress Report section). They will also have access to content and features that provide strategies for engaging in physical activity to aid their quit attempts (refer to the Set and Review Activity Goals section). However, HealthyCells intervention participants will not receive incentives to increase their physical activity.

#### MHBC Intervention: HealthyCells+

In addition to the small financial incentives earned for biochemically verified abstinence, HealthyCells+ intervention participants will earn small incentives for increasing physical activity (ie, daily step count) during their quit attempt. Starting on their scheduled quit date, participants will be rewarded US $0.50 per 1000 steps for every step >4000 daily steps, and they will receive a US $2.50 bonus if they walk at least 6000 steps a day. These criteria were set based on research showing Black adults walk about 4000 steps per day on average [92]. Providing incentives above this threshold will ensure the incentive protocol will not reinforce a sedentary lifestyle, and a bonus for meeting the 6000-step per day goal will reinforce higher levels of daily physical activity and greater positive health benefits [92-100]. Participants will not be incentivized beyond 10,000 steps per day, consistent with previous research showing limited additional health benefits [93,95-100]. Therefore, the maximum amount participants can earn per day for daily steps will be US $5.50 (ie, US $3.00 for 10,000 steps +US $2.50 for step goal bonus), an amount that parallels the amount participants can earn at the fourth week postquit date for daily smoking abstinence.

**Figure 4:**
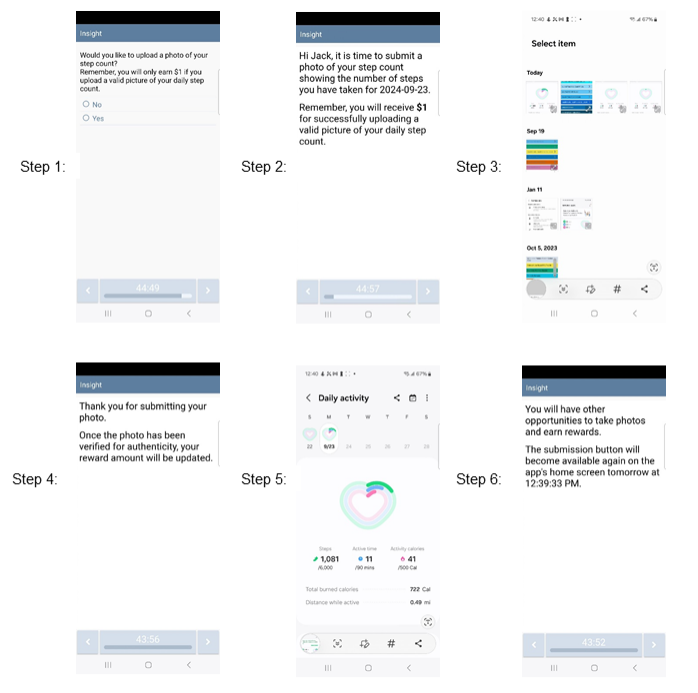
An example of a valid photo upload of activity data to verify daily step count.

Participants will self-report their daily step count via an assessment on the app home menu at the end of each day (based on self-reported typical time to bed). This assessment will also ask participants to upload a picture of their step count (captured by the smartwatch; Figure 4). To promote compliance, participants will receive US $1 for each successful photo upload (up to 7 submissions/US $7/wk). Participants will not earn incentives for their self-reported step count total if an image is not uploaded. Consistent with the incentive schedule for smoking cessation, participants will earn these incentives for increasing physical activity starting on the scheduled quit date up to 4 weeks postquit date. Incentives may also be earned during the final 7 days of the intervention period (eighth wk postquit date). To gain the maximum incentives for increasing physical activity (US $255.5), participants must submit all step count photos and take 10,000 steps daily during all eligible assessment weeks (ie, wk 1-4 and the eighth wk postquit date).

### App Features

#### Overview

**Figure 5:**
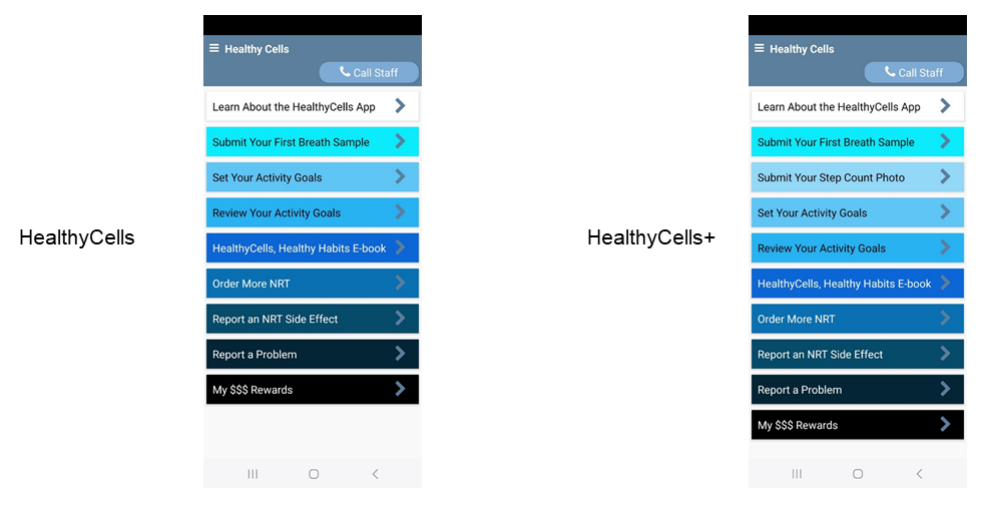
Home screens for HealthyCells (left) and HealthyCells+ (right) apps. NRT: nicotine replacement therapy.

Several app features will be available to help participants earn incentives for abstinence and increase physical activity (Table 2 and Figure 5). Multimedia Appendix 2 provides a complete list of features and a detailed description. A summary of each feature is presented in Table 2.

**Table 2:** Overview of app features.

App features	HealthyCells	HealthyCells+
App Instructions: provide participants with an app overview and study instructions	✓	✓
HealthyCells, Health Habits e-Book: interactive information about smoking and smoking cessation	✓	✓
Breath Sample Submission: submit breath samples using an Bedfont iCOquit Smokerlyzer to verify smoking status and facial recognition software to verify participant identity	✓	✓
Set Activity Goals: set daily steps, active time, and caloric goals	✓	✓
Review Activity Goals: review activity goals and receive feedback to adjust goals for the next day	✓	✓
Submit Step Count Photo: upload an image of activity data for independent verification of step count goals		✓
Report an NRT^a^ Side Effect: report side effects from NRT and receive assistance from study staff	✓	✓
Order More NRT: request more NRT during a quit attempt	✓	✓
Track My Rewards: displays the amount of compensation received for completed study assessments	✓	✓
Report a Problem: report a problem with study equipment or assessments	✓	✓

^a^NRT: nicotine replacement therapy.

#### Learn About the HealthyCells+ App

This feature will be accessible on the home screen at any time during the intervention and will provide an overview of the study apps and instructions for accessing them on their phone.

#### HealthyCells, Healthy Habits E-Book

Participants can access a short e-book to help them prepare for their upcoming quit attempt. Textbox 1 gives an overview of the topics covered in each chapter. On days 3 to 7 of the prequit week, participants will be informed 1 hour before self-reported typical bedtime about the availability of e-chapters. The entire e-book will be accessible on the home screen at any time after the participant’s scheduled quit date.

#### Set and Review Activity Goals

This feature will be accessible on the home screen at the beginning of each day, allowing participants to set activity goals. If a participant inputs a step goal below the recommended amount needed to receive health benefits or incentives, the app will notify them that they have selected an amount below the recommended threshold. Participants can adjust their goals before confirming. Once the participant has set their step goal, this feature will be removed from the home menu screen until the next day.

A total of 8 hours after setting their goal, an option to review their progress will appear on the app home screen. Participants self-report their current step count as indicated on their smartwatch, and the app will automatically calculate whether the participant has met their step goal set earlier in the day. The app will ask participants to rate how challenging it was to meet their current step goal, and their feedback will be used to adjust their step goal for the next day. We created a simple algorithm to set new goals based on the participant’s response dynamically: if they rate their current step goal as “not challenging,” the next day’s step goal will increase by 10%; if “just right,” it will increase by 5%; and if “very challenging,” it will decrease by 10%. For example, if a participant’s goal was 6000 steps and they found it “not challenging,” their new suggested goal would be 6600 steps. Once they review their activity progress, this feature will be unavailable on the home menu until the following day.

#### Report an NRT Side Effect

This feature will be accessible on the home screen at any time, and participants can select from a list of side effects associated with NRT that they experienced during the intervention. Only serious reported side effects will be sent to the study staff and research team via an encrypted email.

#### Order NRT

This feature will be accessible on the home screen at any time, and participants can order up to 4 weeks of additional NRT, which will be sent directly to the participant’s current mailing address.

#### Track My Rewards

Any time during the intervention, participants can track their rewards for submitting breath samples and activity photos (refer to the Study Compensation section) from the app home screen.

#### Report a Problem

From the app home screen, at any time during the intervention, participants can report any issues they have encountered during study participation via an encrypted email sent directly to the research team.

#### Return Study Materials

This menu option will appear on the app home screen after the participant has submitted their final breath sample and uploaded the final photo of their smartwatch activity data to verify their step count. Participants can access instructions about returning their study equipment via study-provided shipping materials.

#### Daily Smoking Cessation Progress Report

Each day during the intervention, participants will receive a notification from their study app 30 minutes after their self-reported wake time to complete a “Daily Smoking Cessation Progress Report.” The app will alert participants visually and audibly for 30 seconds, with a 15-minute snooze option available up to 5 times; after the fifth snooze, the assessment will be marked as missed. The assessment provides feedback on whether smoking status assessments were completed during the previous day, and participants will be asked to self-report confidence in using the Bedfont iCOquit Smokerlyzer and facial recognition. Participants will receive a reminder to use nicotine patches and gum or lozenges daily (or as needed). Participants will also be reminded to wear their smartwatch and set daily activity goals. In addition, participants will be asked to respond to 5 questions assessing sleep quality, mood, stress, energy, and focus.

### Study Compensation

Participants will earn up to US $650 in the HealthyCells condition and up to US $905.5 in the HealthyCells+ condition by earning incentives and completing various study activities (Table 3). Upon completing the enrollment call (ie, the participant is fully eligible and agrees to join the study), participants will receive US $25. Completing the baseline survey (US $50) and interview (US $50) within 72 hours will qualify them for a US $25 bonus, totaling US $125. During the intervention, participants will earn US $1 per breath sample submitted twice daily, with additional bonuses if both samples indicate smoking abstinence (CO <6 ppm), for a possible total of US $325. HealthyCells+ intervention participants will earn US $1 daily for submitting step count photos, with bonuses for exceeding 4000 steps (up to 10,000 total steps) and meeting a 6000-step goal, potentially totaling US $255.5. Completing the postintervention survey at the end of the intervention will offer US $50 and an additional US $50 for participating in the exit interview. If these 2 activities are completed within 72 hours, participants will earn a US $25 bonus, totaling US $125. Participants who return study materials will receive US $25, with an additional US $25 bonus if returned within 7 business days, totaling US $50.

Payments will be delivered via a reloadable GreenPhire ClinCard [86] once per week, with the first payout occurring after the baseline interview and the final payment after study materials are returned. This structured payment system encourages consistent engagement and ensures the timely return of valuable study materials by tying the final payment to their return. Participants can also track their earnings in real time through the app (refer to the Track My Rewards section), which adds transparency to the process.

**Table 3:** Compensation amount and payment schedule for completed study activities.

Activity	Compensation (US $)	Bonus condition	Total compensation (US $)	Study condition	
				HealthyCells^a^	HealthyCells+^b^
Study enrollment^c^	25	None	25	✓	✓
Baseline survey	50	Complete both the baseline survey and interview within 72 h (US $25)	Up to 125	✓	✓
Baseline interview	50	Complete both the baseline survey and interview within 72 h (US $25)	Up to 125	✓	✓
Breath sample submission	1 per sample (2 samples/d)	Not smoking when completing breath samples (up to US $20)	Up to 325	✓	✓
Step count photo Submission	1 per photo (1 upload/d)	Per step above 4000 steps (maximum 10,000 steps; US $3); meeting 6000 step goal (US $2.50)	Up to 255.5		✓
Postintervention survey	50	Complete both the postintervention survey and baseline survey within 72 h (US $25)	Up to 125	✓	✓
Exit interview	50	Complete both the postintervention survey and baseline survey within 72 h (US $25)	Up to 125	✓	✓
Return study materials	25	Return study materials within 7 business days (US $25)	Up to 50	✓	✓

^a^The total compensation was up to US $650.

^b^The total compensation was up to US $905.5.

^c^Participants only earn US $25 if they successfully enroll in the study.

### Postintervention Assessment and Treatment Outcomes

At the eighth week postquit date, participants will complete a postintervention assessment via REDCap [85], answering questions similar to those in the baseline assessment to track changes in physical and mental health and health behaviors and to assess app impressions (Multimedia Appendix 1). A final semistructured interview will gather feedback on the app and suggestions for improvement (Table 1). The primary outcome for smoking cessation will be CO-verified, 7-day point prevalence smoking abstinence at 8 weeks postquit date, which will be assessed via smartphone-based self-report and a corroborating CO assessment. Expired CO is a valid indicator of smoking and cessation and compares favorably with cotinine and other biochemical measures [101]. Daily abstinence will be examined as a secondary outcome and defined as self-reported abstinence within the day combined with 2 breath CO sample submissions <6 ppm and identity verification. The number of days and consecutive days of CO-verified abstinence will be explored as additional outcomes. The other primary outcome will be the net change in average daily steps during the eighth week postquit date compared with average daily steps the week before the quit attempt (HealthyCells+ group only). Second, we will explore the number of total and consecutive days that ≥6000 steps were achieved as additional outcomes [102]. A valid day of observation for daily steps will be defined as (1) a smartwatch that was worn for at least 10 waking hours with (2) ≥500 steps taken within a day [103].

To ensure accurate, objective measurement of step count, study staff will pair each participant’s Samsung smartwatch with a Samsung smartphone and create a unique Samsung Health account before shipping the study materials. The Samsung Health app integrates seamlessly with the smartwatch, allowing continuous step count tracking. These data sync automatically from the watch to the app, enabling real-time data capture. Throughout the intervention, the research team will periodically access each participant’s Samsung Health account to securely export step data, which will be saved as a file for analysis. Once participants return the phone and smartwatch, the study team will directly extract step data from the smartwatch to verify and complement the app-synced step data. This dual extraction process enhances data reliability, ensuring comprehensive and objective measurement of participants’ physical activity [102]. After this extraction, the phone and smartwatch will be reset to factory settings to prepare them for use with another participant, ensuring privacy and consistency across participants.

### Feasibility and Engagement Outcomes

We will assess several metrics to gauge the feasibility of evaluating the intervention in a larger-scale trial. Key metrics include completion rates for surveys and interviews (retention and engagement), instances of lost or damaged phones or watches (technology use barriers), and participant engagement with app features, such as completing the e-book for smoking cessation and setting and reviewing daily step goals. We will also characterize the frequency of photo uploads of daily step counts, completion of daily smoking status assessments, NRT adherence, completion of counseling sessions, and duration of smartwatch wear. These feasibility metrics will also be explored qualitatively by interviewing participants about their experiences with the intervention (ie, exit interview), such as their engagement with app features, ease of completing assessments, and any challenges encountered with study equipment.

### Sample Size Justification

The sample size of 60 participants was chosen based on recommendations for feasibility trials [104,105], which prioritize assessing study processes, recruitment, retention, and intervention engagement rather than detecting statistically significant effects. This sample size is sufficient to evaluate key feasibility metrics, such as recruitment rates (target: ≥5 participants/mo), retention (≥75% completion), and adherence to intervention components, which are critical for planning a fully powered randomized controlled trial. In addition, this sample size allows for preliminary comparisons between the HealthyCells and HealthyCells+ groups to explore trends in CO-verified smoking abstinence and changes in daily step counts.

### Analytic Plan

This analytic plan will focus on describing intervention feasibility. Feasibility indicators will include enrollment of all study participants within 12 months (≥5 adults enrolled/mo), retention of at least 75% of the participants at 8 weeks postquit date, achieving 20% smoking abstinence at 8 weeks postquit date (consistent with NRT and behavioral support standards) [104,105], and observing a meaningful 500 to 1500 [106,107] average daily step increase at eighth week postquit date. SAS (version 9.4; SAS Institute) [108] will be used for all statistical analyses, generating descriptive statistics to compare demographics, smoking status, and physical activity levels by treatment group. Group comparisons on participant characteristics and outcomes will use 2-tailed *t* tests for continuous variables and chi-square tests for categorical variables. Given the small sample size and pilot design (N=60), we will report all potential group differences with the α set at *P*<.20, following recommendations for pilot studies [109].

Smoking cessation will be analyzed in two ways: (1) an intention-to-treat analysis with missing data classified as smoking and (2) a completers-only analysis excluding participants with missing data. For physical activity outcomes, specifically the change in average daily step count from the baseline week to the eighth week postquit date, *t* tests will evaluate pre-post differences between groups. Days with <500 steps or <10 hours of watch wear time (indicating nonwear time or recording error) will be excluded. Participants must have at least 4 days of valid step count data to be included in the analyses.

Exploratory analyses will examine the association between physical activity and smoking cessation success using continuous and categorical approaches, with 6000 steps as the threshold for categorical analyses. Logistic regression analysis will evaluate the impact of average daily step count based on all postquit assessment weeks (5 wk in total) on CO-verified 7-day point prevalence abstinence at 8 weeks postquit date. In addition, participants will be categorized based on achieving a daily average of ≥6000 steps, allowing us to evaluate whether higher activity levels (≥6000 steps) are associated with greater cessation success. Finally, we will explore additional metrics, including the number of consecutive abstinent days and the frequency of meeting daily step goals. The analysis will examine these metrics as continuous and categorical variables to assess their association with smoking cessation rates.

### Qualitative Analysis Plan

The baseline and exit interviews will be evaluated separately to comprehensively understand participants’ experiences and impressions at different stages of the mHealth intervention. Transcripts from both interviews will be analyzed in NVivo (version 12; Lumivero) using an inductive thematic approach [110,111]. A codebook will be developed collaboratively by coding a subset of interviews, with iterative refinements to ensure reliability before the lead author’s independent coding of the remaining transcripts. Periodic team reviews will resolve discrepancies.

The baseline interview will assess participants’ initial perspectives and familiarity with mHealth tools, which may inform their engagement with the intervention. Key focus areas will include motivation for participating in mHealth research, smartphone use habits, and previous experiences with health apps and Bluetooth-enabled devices. By examining these factors, the analysis will capture participants’ baseline comfort levels with digital health tools, previous exposure to apps for smoking cessation and physical activity, and any initial preferences for culturally tailored health behavior interventions within the Black population. Insights from these domains will provide context for interpreting participants’ engagement with the intervention and potential barriers that could influence adherence.

The exit interview will assess participants’ experiences with the intervention, focusing on perceptions of the cultural tailoring and usability of the intervention components. A central focus will be feedback on the e-book modeled after *Pathways to Freedom* [48], with analysis capturing participants’ impressions of its cultural relevance, clarity, and perceived impact on smoking cessation. Participants’ feedback on which sections resonated, any challenges encountered, and suggestions for improvement will provide insights into enhancing this culturally tailored component. Further domains in the exit interview will evaluate participants’ overall impressions of the app features, experiences with the smartwatch, adherence to NRT, and any technical issues encountered with study equipment. This analysis aims to identify strengths and weaknesses of the intervention from the user perspective, including insights into specific app features that supported or hindered engagement and practical challenges with using the devices.

We will synthesize findings from the baseline and exit interviews to identify overarching themes related to engagement, cultural resonance, and barriers to adherence. This holistic analysis will reveal how initial expectations and previous experiences influenced participant experiences and how the intervention can be refined to improve acceptability and effectiveness for larger-scale studies. Examining recurring themes across interviews may provide actionable insights for enhancing intervention design, especially regarding cultural tailoring, usability, and support for behavior change.

### Integration of Study Findings

We will use a mixed methods approach to capture a holistic view of intervention feasibility and participants’ experiences [112]. Quantitative data will provide intervention outcomes and milestones, and qualitative findings will uncover insights into participants’ expectations, experiences, and perceptions regarding the intervention. Triangulation will occur through a systematic comparison of quantitative metrics with qualitative themes [112,113]. For example, if quantitative results indicate high smoking cessation rates, qualitative data will be examined to identify participant-reported factors that facilitated this success. Conversely, if step count improvements are minimal, qualitative insights may help reveal barriers to physical activity engagement or limitations within the app that could be addressed in future studies. This combined approach will provide information about feasibility and generate participant-driven insights, increasing the likelihood that future interventions will be effective and tailored to meet community needs.

## Results

**Figure 6:**
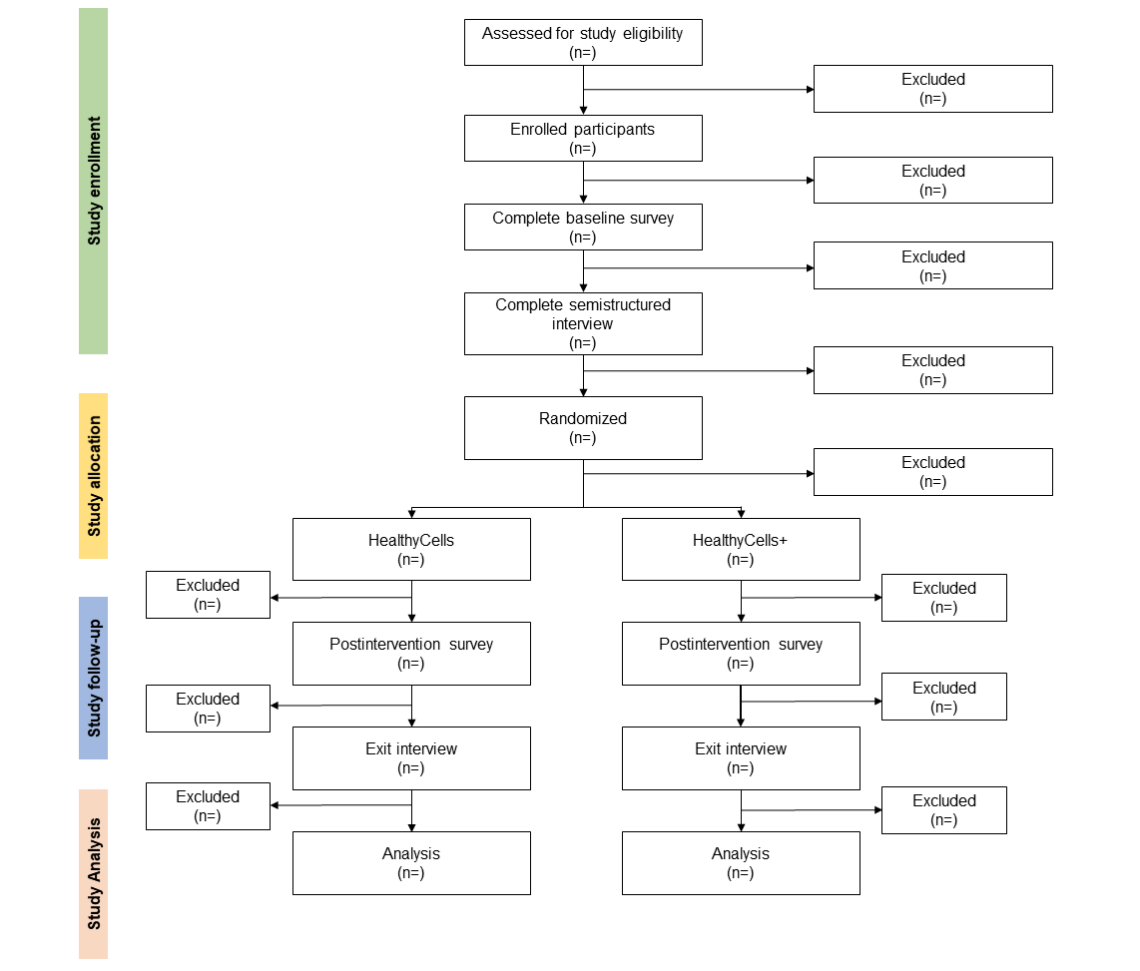
Participant recruitment, allocation, intervention adherence, and follow-up in the feasibility randomized controlled trial evaluating the HealthyCells interventions for smoking cessation and physical activity promotion (CONSORT [Consolidated Standards of Reporting Trials] flow diagram).

This study was reviewed and funded by the National Institute of Minority Health and Health Disparities (Multimedia Appendix 3). This study was approved by the institutional review board of the University of Oklahoma Health Sciences Center (protocol #14094) on 12/10/2021. Data collection is expected to start in February 2025 and finish in September 2025 (Figure 6 depicts the CONSORT [Consolidated Standards of Reporting Trials] diagram). Primary data are expected to be analyzed by October 2025. The preparation of manuscripts on primary and secondary outcomes is expected to start in late 2025 and early 2026.

## Discussion

### Principal Findings

This study evaluates the feasibility of the HealthyCells intervention, an app-based mHealth approach integrating financial incentives for smoking cessation and physical activity promotion among Black adults. We anticipate that participants in the HealthyCells+ group, who receive incentives for both smoking abstinence and physical activity, will achieve greater increases in daily step counts and higher rates of CO-verified smoking abstinence compared to the HealthyCells group, who are incentivized for smoking abstinence only. Feasibility metrics, including recruitment, retention, and participant adherence, are expected to demonstrate strong engagement and acceptability of the intervention over the 9-week study period.

### Comparison to Prior Work

This study builds upon previous MHBC mHealth interventions, such as See Me Smoke-Free [114] and PhoS [115], which showed potential to promote behavior change but were limited by reliance on self-reported outcomes and low adherence rates [46,116,117]. In contrast, HealthyCells incorporates objective measures of behavior (eg, CO breath samples for smoking abstinence and step counts verified by smartwatches), addressing these limitations. In addition, culturally relevant content, such as that inspired by *Pathways to Freedom* [48,49], distinguishes HealthyCells from other interventions by tailoring messages to resonate specifically with Black adults. This study also advances CM research by combining financial incentives for multiple behaviors, a strategy that has shown promise in previous studies [34-38].

### Strengths and Limitations

A key strength of this study is its focus on a population disproportionately affected by tobacco- and obesity-related health disparities. By leveraging mHealth technology and CM, this intervention offers a scalable and accessible approach to behavior change. Objective tracking of outcomes ensures data reliability, and the inclusion of culturally tailored content enhances the potential for participant engagement.

However, this study has limitations inherent to feasibility trials. The sample size (n=60) is small and not powered to detect statistically significant differences in outcomes. In addition, the absence of a control group limits conclusions about the intervention’s effectiveness. Despite these limitations, this study will provide critical pilot data for a fully powered randomized controlled trial.

### Future Directions

Findings from this feasibility study will guide the refinement of the HealthyCells intervention for larger-scale trials. Future research will explore the effects of long-term interventions, the maintenance of behavior change after incentives are withdrawn, and the scalability of interventions to other underserved populations. In addition, further refinements to the app based on participant feedback will aim to enhance usability and engagement. Ultimately, this research has the potential to contribute to health equity by addressing smoking and physical inactivity in high-risk populations using innovative, culturally tailored, and technology-driven strategies.

## Appendix

Multimedia Appendix 1 Overview of study measures across each assessment type.

Multimedia Appendix 2 Overview of scheduled assessment and menu options available in both study apps.

Multimedia Appendix 3 National Institute of Health peer-review report.

## Abbreviations

CM: contingency management
CO: carbon monoxide
CONSORT: Consolidated Standards of Reporting Trials
MHBC: multiple health behavior change
mHealth: mobile health
NRT: nicotine replacement therapy
REDCap: Research Electronic Data Capture

## Data Availability

The datasets generated and analyzed during this study will be made publicly available in a suitable data repository following the publication of all primary and secondary analyses. Until that time, the data will be available from the corresponding author on reasonable request.
