# Supplementary material for: An mHealth Intervention With Financial Incentives to Promote Smoking Cessation and Physical Activity Among Black Adults: Protocol for a Feasibility Randomized Controlled Trial
Source: JMIR Res Protoc. 2025 Jan 31;14:e69771. doi: 10.2196/69771 (PMC11829183; doi:10.2196/69771)
Supplement: Multimedia Appendix 1 [file resprot_v14i1e69771_app1.docx]

| **Multimedia Appendix 1.** Overview of study measures across each assessment type | | | | | | | | |
| --- | --- | --- | --- | --- | --- | --- | --- | --- |
| **Descriptive variable** | **No. of items** | **The full battery of assessments** | | | | | | |
|  |  | Screening survey | Enrollment  call | Baseline survey | Baseline interview | App set-up | Post-intervention survey | Exit- interview |
| Screening questionnaire | 48 | X |  |  |  |  |  |  |
| Enrollment questionnaire[1, 2] | 80 |  | X |  |  |  |  |  |
| Sociodemographic characteristics[3] | 62 |  |  | X |  |  |  |  |
| Smoking history questionnaire[4-10] | 102 |  |  | X |  |  | X |  |
| Health behavior, mental and physical health questionnaire[11-22] | 163 |  |  | X |  |  | X |  |
| Psychosocial factors questionnaire[23-32] | 158 |  |  | X |  |  | X |  |
| App impressions[33, 34] | 58 |  |  |  |  |  | X |  |
| Baseline interview questionnaire | 59 |  |  |  | X |  |  |  |
| App-setup call questionnaire | 19 |  |  |  |  | X |  |  |
| Exit interview questionnaire | 90 |  |  |  |  |  |  | X |
| **Total number of items** |  | 48 | 80 | 493 | 59 | 19 | 481 | 90 |
| **Type of assessment** |  | Self-administered | Interviewer-administered | Self-administered | Interviewer-administered | Interviewer-administered | Self-administered | Interviewer-administered |
| **Total time needed to complete the assessment (minutes)** |  | 15 – 20 | 30 – 60 | 60 – 90 | 60 – 90 | 30 – 60 | 60 – 90 | 60 – 90 |

**REFERENCES**

[1] P. H. Lee, D. J. Macfarlane, T. H. Lam, and S. M. Stewart, "Validity of the international physical activity questionnaire short form (IPAQ-SF): A systematic review," *International Journal of Behavioral Nutrition and Physical Activity,* vol. 8, no. 1, p. 115, 2011/10/21 2011, doi: 10.1186/1479-5868-8-115.

[2] A. M. Arozullah *et al.*, "Development and validation of a short-form, rapid estimate of adult literacy in medicine," (in eng), *Med Care,* vol. 45, no. 11, pp. 1026-33, Nov 2007, doi: 10.1097/MLR.0b013e3180616c1b.

[3] N. Adler, E. Epel, G. Castellazzo, and J. Ickovics, "MacArthur scale of subjective social status–Adult version," ed, 2000.

[4] J. F. Etter, M. M. Bergman, J. P. Humair, and T. V. Perneger, "Development and validation of a scale measuring self-efficacy of current and former smokers," (in eng), *Addiction,* vol. 95, no. 6, pp. 901-13, Jun 2000, doi: 10.1046/j.1360-0443.2000.9569017.x.

[5] J. Emery *et al.*, "Development and Content Validation of a Questionnaire for Measuring Beliefs About Using Nicotine Replacement Therapy for Smoking Cessation in Pregnancy," (in eng), *Nicotine Tob Res,* vol. 25, no. 7, pp. 1310-1318, Jun 9 2023, doi: 10.1093/ntr/ntad030.

[6] T. R. Schlam *et al.*, "Can we increase smokers' adherence to nicotine replacement therapy and does this help them quit?," (in eng), *Psychopharmacology (Berl),* vol. 235, no. 7, pp. 2065-2075, Jul 2018, doi: 10.1007/s00213-018-4903-y.

[7] T. F. Heatherton, L. T. Kozlowski, R. C. Frecker, and K. O. Fagerström, "The Fagerström Test for Nicotine Dependence: a revision of the Fagerström Tolerance Questionnaire," (in eng), *Br J Addict,* vol. 86, no. 9, pp. 1119-27, Sep 1991, doi: 10.1111/j.1360-0443.1991.tb01879.x.

[8] K. C. Wheeler, K. E. Fletcher, R. J. Wellman, and J. R. Difranza, "Screening adolescents for nicotine dependence: the Hooked On Nicotine Checklist," *Journal of Adolescent Health,* vol. 35, no. 3, pp. 225-230, 2004.

[9] S. S. Smith, M. E. Piper, D. M. Bolt, J. T. Kaye, M. C. Fiore, and T. B. Baker, "Revision of the Wisconsin Smoking Withdrawal Scale: Development of brief and long forms," (in eng), *Psychol Assess,* vol. 33, no. 3, pp. 255-266, Mar 2021, doi: 10.1037/pas0000978.

[10] F. Guay, R. J. Vallerand, and C. Blanchard, "On the assessment of situational intrinsic and extrinsic motivation: The Situational Motivation Scale (SIMS)," *Motivation and emotion,* vol. 24, pp. 175-213, 2000.

[11] C. L. Cleland, R. F. Hunter, F. Kee, M. E. Cupples, J. F. Sallis, and M. A. Tully, "Validity of the Global Physical Activity Questionnaire (GPAQ) in assessing levels and change in moderate-vigorous physical activity and sedentary behaviour," *BMC Public Health,* vol. 14, no. 1, p. 1255, 2014/12/10 2014, doi: 10.1186/1471-2458-14-1255.

[12] D. E. Rosenberg, G. J. Norman, N. Wagner, K. Patrick, K. J. Calfas, and J. F. Sallis, "Reliability and validity of the Sedentary Behavior Questionnaire (SBQ) for adults," (in eng), *J Phys Act Health,* vol. 7, no. 6, pp. 697-705, Nov 2010, doi: 10.1123/jpah.7.6.697.

[13] B. Resnick and L. S. Jenkins, "Testing the reliability and validity of the Self-Efficacy for Exercise scale," (in eng), *Nurs Res,* vol. 49, no. 3, pp. 154-9, May-Jun 2000, doi: 10.1097/00006199-200005000-00007.

[14] J. F. Sallis *et al.*, "Evaluating a brief self-report measure of neighborhood environments for physical activity research and surveillance: Physical Activity Neighborhood Environment Scale (PANES)," (in eng), *J Phys Act Health,* vol. 7, no. 4, pp. 533-40, Jul 2010, doi: 10.1123/jpah.7.4.533.

[15] E. Snyder, B. Cai, C. DeMuro, M. F. Morrison, and W. Ball, "A New Single-Item Sleep Quality Scale: Results of Psychometric Evaluation in Patients With Chronic Primary Insomnia and Depression," (in eng), *J Clin Sleep Med,* vol. 14, no. 11, pp. 1849-1857, Nov 15 2018, doi: 10.5664/jcsm.7478.

[16] M. P. Jensen and C. A. McFarland, "Increasing the reliability and validity of pain intensity measurement in chronic pain patients," *Pain,* vol. 55, no. 2, pp. 195-203, 1993.

[17] K. Kroenke, R. L. Spitzer, and J. B. Williams, "The PHQ-9: validity of a brief depression severity measure," (in eng), *J Gen Intern Med,* vol. 16, no. 9, pp. 606-13, Sep 2001, doi: 10.1046/j.1525-1497.2001.016009606.x.

[18] R. L. Spitzer, K. Kroenke, J. B. Williams, and B. Löwe, "A brief measure for assessing generalized anxiety disorder: the GAD-7," (in eng), *Arch Intern Med,* vol. 166, no. 10, pp. 1092-7, May 22 2006, doi: 10.1001/archinte.166.10.1092.

[19] M. J. Zvolensky *et al.*, "Refinement of anxiety sensitivity measurement: The Short Scale Anxiety Sensitivity Index (SSASI)," (in eng), *Psychiatry Res,* vol. 269, pp. 549-557, Nov 2018, doi: 10.1016/j.psychres.2018.08.115.

[20] L. Richard, M. Emily, R. Heidi, K. Dean, F. Matthew, and C. Michelle, "Dimensional assessment of posttraumatic stress disorder in DSM-5," *Psychiatry Research,* vol. 218, no. 1, pp. 143-147, 2014, doi: https://doi.org/10.1016/j.psychres.2014.03.032.

[21] D. L. Steinweg and H. Worth, "Alcoholism: the keys to the CAGE," *The American journal of medicine,* vol. 94, no. 5, pp. 520-523, 1993.

[22] S. J. Adamson *et al.*, "An improved brief measure of cannabis misuse: the Cannabis Use Disorders Identification Test-Revised (CUDIT-R)," *Drug and alcohol dependence,* vol. 110, no. 1-2, pp. 137-143, 2010.

[23] W. E. Mehling, M. Acree, A. Stewart, J. Silas, and A. Jones, "The Multidimensional Assessment of Interoceptive Awareness, Version 2 (MAIA-2)," (in eng), *PLoS One,* vol. 13, no. 12, p. e0208034, 2018, doi: 10.1371/journal.pone.0208034.

[24] S. Cohen, T. Kamarck, and R. Mermelstein, "A global measure of perceived stress," *Journal of health and social behavior,* pp. 385-396, 1983.

[25] M. E. Hughes, L. J. Waite, L. C. Hawkley, and J. T. Cacioppo, "A short scale for measuring loneliness in large surveys: Results from two population-based studies," *Research on aging,* vol. 26, no. 6, pp. 655-672, 2004.

[26] D. R. Williams, Y. Yu, J. S. Jackson, and N. B. Anderson, "Racial differences in physical and mental health: Socio-economic status, stress and discrimination," *Journal of health psychology,* vol. 2, no. 3, pp. 335-351, 1997.

[27] V. J. Felitti *et al.*, "Relationship of childhood abuse and household dysfunction to many of the leading causes of death in adults: The Adverse Childhood Experiences (ACE) Study," *American journal of preventive medicine,* vol. 14, no. 4, pp. 245-258, 1998.

[28] R. E. Davis *et al.*, "A new audience segmentation tool for African Americans: the black identity classification scale," (in eng), *J Health Commun,* vol. 15, no. 5, pp. 532-54, Jul 2010, doi: 10.1080/10810730.2010.492563.

[29] S. Huber and O. W. Huber, "The centrality of religiosity scale (CRS)," *Religions,* vol. 3, no. 3, pp. 710-724, 2012.

[30] M. A. Chesney, T. B. Neilands, D. B. Chambers, J. M. Taylor, and S. Folkman, "A validity and reliability study of the coping self-efficacy scale," (in eng), *Br J Health Psychol,* vol. 11, no. Pt 3, pp. 421-37, Sep 2006, doi: 10.1348/135910705x53155.

[31] C. D. Sherbourne and A. L. Stewart, "The MOS social support survey," (in eng), *Soc Sci Med,* vol. 32, no. 6, pp. 705-14, 1991, doi: 10.1016/0277-9536(91)90150-b.

[32] S. Aktay, "A validity and reliability study of the smartphone self-efficacy scale," *International Technology and Education Journal,* vol. 2, no. 2, pp. 11-18, 2018.

[33] A. Bangor, P. T. Kortum, and J. T. Miller, "An empirical evaluation of the system usability scale," *Intl. Journal of Human–Computer Interaction,* vol. 24, no. 6, pp. 574-594, 2008.

[34] H. L. O’Brien, P. Cairns, and M. Hall, "A practical approach to measuring user engagement with the refined user engagement scale (UES) and new UES short form," *International Journal of Human-Computer Studies,* vol. 112, pp. 28-39, 2018.
