# Supplementary material for: An mHealth Intervention With Financial Incentives to Promote Smoking Cessation and Physical Activity Among Black Adults: Protocol for a Feasibility Randomized Controlled Trial
Source: JMIR Res Protoc. 2025 Jan 31;14:e69771. doi: 10.2196/69771 (PMC11829183; doi:10.2196/69771)
Supplement: Multimedia Appendix 2 [file resprot_v14i1e69771_app2.docx]

| **Multimedia Appendix 2.** Overview of scheduled assessment and menu options available in both study apps. | | | | | |
| --- | --- | --- | --- | --- | --- |
| **Name of Assessment** | **Brief Description** | **Assessment Type** | **Study Condition** | | **Time Window** |
|  |  |  | HealthyCells | HealthyCells+ |  |
| Learn About the HealthyCells App | This is an assessment that provides participants with an overview of the HealthyCells app. | On-demand^a^ | X | X |  |
| HealthyCells, Health Habits E- Book | An assessment that allows participants to review 1 of 5 learning modules on smoking cessation. | Scheduled assessment^b^ | X | X | One learning module is sent to participants 2 hours before bedtime during Day 3, Day 4, Day 5, Day 6, and Day 7 of the pre-quit week. All learning modules appear on the app home menu under the title “HealthyCells, Health Habits E-Book” after participants’ quit date. |
| Submit Breath Sample | This assessment allows participants to submit a breath sample using a Bedfont iCOquit® device to verify their smoking status and use facial recognition software to verify their identity. | Scheduled assessment^b^ | X | X | It can be completed twice daily. The first assessment is available all day, and the second is only available 8 hours after the first assessment is completed. These assessments appear on the home menu. Upon completion, each assessment no longer appears on the home menu until the next day. |
| Set Activity Goals | This assessment allows participants to set daily steps, active time, and caloric goals. | On-demand^a^ | X | X | Upon completion, the assessment no longer appears on the home menu until the next day. |
| Review Activity Goals | This is an assessment that allows participants to review their daily activity goals and receive feedback to adjust goals for the next day. | Scheduled assessment^b^ | X | X | Participants must complete “Set Your Activity Goals” to activate this assessment. It becomes available on the home menu 8 hours after the completion of “Set Your Activity Goals.” Once completed, it no longer appears on the home menu until the next day. |
| Submit Step Count Photo | This is an assessment that allows participants to upload an image of smartwatch activity data for independent verification of step count goals. | Scheduled assessment^b^ |  | X | Available once per day. This assessment appears on the home menu. Upon completion, the assessment no longer appears on the home menu until the next day. |
| Report an NRT Side Effect | This assessment allows participants to report side effects from NRT and receive assistance from study staff. | On-demand^a^ | X | X |  |
| Order NRT | This assessment allows participants to order more NRT during their quit attempt. | On-demand^a^ | X | X |  |
| Report a Problem | An assessment that allows participants to report app or study-related problems directly to the study team | On-demand^a^ | X | X |  |
| Track My $$$ Rewards | An assessment that allows participants to track the rewards they have earned throughout study participation. | On-Demand^b^ | X | X |  |
| Welcome Message | An assessment that provides an overview of the assigned study app and its associated features. | Scheduled assessment^b^ | X | X | Day 1 during the pre-quit week and Day 1 of the first week of the quit attempt. Sent to participants 2 hours after wake time. |
| Daily Smoking Cessation Progress Report | An assessment that provides personalized feedback based on the breath sample(s) submitted on the previous day. | Scheduled assessment^b^ | X | X | Assessment is sent once per day, 2 hours after wake time. |
| Return Study Materials | An assessment that serves as a checklist to return the equipment loaned to the participant for study participation. | On-Demand^b^ | X | X | This assessment is only available at the end of Week 8 after the participant has submitted their final breath sample and uploaded the final photo of smartwatch activity data to verify the step count. |
| ^a^ **On-demand** content is accessible on the app home menu. Most on-demand content is available at any time once the intervention starts, other on-demand content is unlocked as the participant progresses through the study.  ^b^ A **scheduled assessment** is triggered by the study-provided smartphone. The phone will audibly and visually cue these surveys for 30 seconds. If a participant does not respond after five prompts, the assessment will be recorded as missed. Some scheduled assessments may also appear on the app home menu if certain conditions are met. | | | | | |
