# Supplementary material for: An mHealth Intervention With Financial Incentives to Promote Smoking Cessation and Physical Activity Among Black Adults: Protocol for a Feasibility Randomized Controlled Trial
Source: JMIR Res Protoc. 2025 Jan 31;14:e69771. doi: 10.2196/69771 (PMC11829183; doi:10.2196/69771)
Supplement: Multimedia Appendix 3 [file resprot_v14i1e69771_app3.pdf]

SUMMARY STATEMENT
(Privileged Communication)

PROGRAM CONTACT:
Jennifer Alvidrez
301-594-9567
alvidrezjl@mail.nih.gov

Release Date: **12/14/2020**
Revised Date: **12/14/2020**

Application Number: **1 K01 MD015295-01A1**
Formerly: **1K01MD015295-01**

Principal Investigator

ALEXANDER, ADAM C

Applicant Organization: UNIVERSITY OF OKLAHOMA HLTH SCIENCES CTR

Review Group: **ZMD1 DRI (J1)**
National Institute on Minority Health and Health Disparities Special Emphasis Panel
NIMHD K01 Career Development Award Peer Review

Meeting Date: **11/09/2020**
Council: **JAN 2021**
Requested Start: **04/01/2021**
RFA/PA: **PA20-176**
PCC: **CPS12JA**
Dual IC(s): **HL**

Project Title: **HealthyCells: A Culturally-Tailored Smoking Cessation Smartphone Intervention for African Americans with Adjunctive Treatment for Sedentary Behavior**
SRG Action: **Impact Score:31**
Next Steps: **Visit https://grants.nih.gov/grants/next_steps.htm**
Human Subjects: 30-Human subjects involved - Certified, no SRG concerns
Animal Subjects: 10-No live vertebrate animals involved for competing appl.
Gender: 1A-Both genders, scientifically acceptable
Minority: 2A-Only minorities, scientifically acceptable
Age: 3A-No children included, scientifically acceptable

Project Year	Direct Costs Requested	Estimated Total Cost
1	123,187	132,994
2	123,393	133,217
3	124,735	134,666
4	124,736	134,667
5	124,736	134,667
TOTAL	620,787	670,210

ADMINISTRATIVE BUDGET NOTE: The budget shown is the requested budget and has not been adjusted to reflect any recommendations made by reviewers. If an award is planned, the costs will be calculated by Institute grants management staff based on the recommendations outlined below in the COMMITTEE BUDGET RECOMMENDATIONS section.

1K01MD015295-01A1 Alexander, Adam

RESUME AND SUMMARY OF DISCUSSION: This application is resubmitted by the University of Oklahoma Health Sciences, Oklahoma City, in response to PA-20-176: Mentored Research Scientist Development Award (Parent K01 – Independent Clinical Trial Required). The candidate is a second-year postdoctoral research fellow at the Tobacco Research Center (TRC) with a PhD in social and behavioral sciences. His research focuses on smoking cessation and reducing sedentary behavior among African Americans using evidence-based, culturally tailored smartphone interventions. This application has many strengths. The candidate is very strong with a record of publications in relevant areas of research. The career development plan is well-designed and includes an excellent individualized learning plan with structured coursework that maps onto the research plan and benchmarks for progress. The research plan involves the integration of two existing phone apps and the addition of cultural tailoring for Black smokers to promote cessation and increase activity. The timeline seems reasonable. The mentorship team is strong with relevant expertise and strong support for the candidate. The environment is outstanding with numerous resources that will provide an excellent training opportunity for the candidate. Evidence of institutional support for the candidate is shown in the recent appointment to Assistant Professor. The resubmitted application is considered mostly responsive to prior reviews. Minor addressable weaknesses are noted. In the research plan, the approach does not unfold in clear steps from formative input to app build to testing, data collection, and analysis. The analysis plan needs to reflect relevant detail. More description of TRC protocols and resources available for the app development process would be helpful, including participant recruitment, collection of user feedback, and intervention testing. It is unclear why aims are reduced from three to two; aims typically reflect the steps outlined in the approach. For revised Aim 1, a literature review is used to inform the cultural tailoring of content and user relevance without obtaining formative input from end users regarding content prior to app development. The focus on sedentary behavior is not addressed. The proposed app's interactive design seems fairly complicated, but technical assistance for end users is not addressed. For testing of intervention efficacy, the change to a pre- and post-test design does not address the need to still confirm intervention efficacy in a larger comparison trial. Overall, the panel expressed enthusiasm for this promising candidate who has a high likelihood of achieving an independent research career. This application is rated as excellent with some minor weaknesses and is likely to have high overall impact.

DESCRIPTION (provided by applicant): Successfully increasing cessation rates among African Americans would reduce the racial disparity in mortality between African Americans and Whites by up to 20%. Many smoking cessation interventions include physical activity components to boost cessation rates, but no studies have attempted to promote sedentary behavior reduction as a cessation strategy. Further, smartphones can facilitate intensive behavioral interventions without requiring burdensome in-person treatment, and these interventions can be tailored to address the specific needs of African Americans and capitalize on cultural strengths to reduce barriers and encourage behavior change. Our research team has developed numerous smartphone interventions to address a range of modifiable health risk factors among vulnerable populations, which are capable of being culturally tailored for African Americans. Of relevance to this research proposal, one is a smartphone-based contingency management intervention that provides financial rewards for smoking abstinence after remote verification of smoking status using low-cost portable carbon monoxide monitors and facial recognition software (i.e., PrevailGO). The other is a smartphone-based sedentary reduction intervention that uses wrist-worn activity monitors in combination with smartphone technology to monitor activity in real-time and deliver activity prompts when individuals are engaged in prolonged bouts of sedentary behavior (i.e., SMARTpath). My long-term goal is to improve smoking cessation outcomes among African Americans using evidence-based, culturally tailored smartphone interventions. The overall objectives of this research proposal, which is the next step toward achieving this long-term goal, are to (a) create a smartphone application for smoking cessation (i.e., HealthyCells), which will be achieved by integrating two pre-existing evidence-based smartphone interventions, PrevailGO and SMARTpath, (b) create

culturally tailored treatment content (i.e., messages, images, and videos) for African American smokers, and (c) evaluate the feasibility of HealthyCells at addressing smoking and sedentary behavior among African Americans. A pilot sample of African American smokers (N = 15) will briefly use the HealthyCells app and provide critical feedback through semi- structured interviews to refine the smartphone intervention. Once the HealthyCells app is refined, African Americans (N = 30) who are interested in quitting smoking will use the app during a scheduled quit attempt. Starting on the scheduled quit date, HealthyCells will prompt participants to complete twice-daily remote smoking status assessments to earn rewards for abstinence. The app will deliver real-time messages telling participants to stand up and move around during prolonged bouts of sedentary behavior (i.e., ≥ 30 minutes of uninterrupted time spent in a sitting, reclining, or lying posture). Participants will also have on-demand access to culturally tailored information and strategies for remaining abstinent and reducing sedentary behavior within the HealthyCells app. The primary outcomes will be biochemically confirmed point prevalence smoking abstinence at 8 weeks post-quit date, and the difference in sedentary time 7 consecutive days before quitting compared with 7 consecutive days at 8-weeks post-quit, as measured by a research- grade accelerometer.

PUBLIC HEALTH RELEVANCE: Successfully increasing cessation rates among African Americans will reduce the racial disparity in mortality among African Americans and Whites by up to 20%. This K01 research proposal outlines, in detail, a plan to develop an evidence-based smartphone intervention for smoking cessation with adjunctive sedentary behavior reduction, which will be culturally tailored for African Americans. Although there are numerous in-person interventions for smoking cessation that have been tailored for African Americans and minorities, smartphone-based interventions are still lacking; thus, this proposal represents an innovative and modern approach for eliminating tobacco-related health disparities in this underserved population.

CRITIQUES: The written critiques of individual reviewers are provided in essentially unedited form below. These critiques were prepared prior to the meeting and may not have been revised afterwards. The “Resume and Summary of Discussion” above summarizes the final opinions of the committee.

CRITIQUE 1

Candidate: 1
Career Development Plan/Career Goals /Plan to Provide Mentoring: 1
Research Plan: 3
Mentor(s), Co-Mentor(s), Consultant(s), Collaborator(s): 1
Environment Commitment to the Candidate: 1

OVERALL IMPACT: In the United States, black Americans are at increased risk for heart disease and experience disproportionate mortality from the illness compared to the general population. A Higher rate of tobacco smoking among African Americans is a major contributor to this disparity. Therefore, interventions that can effectively lower smoking prevalence in the African American population are needed. This K01 application proposes research that includes the development of a cell phone application” (HealthyCells) that will promote quitting among African American smokers. Interventions of this sort that can be applied outside the clinical setting are important for addressing race/ethnic disparities in health outcomes. The tailoring of the quit smoking content in HealthyCells specifically to African Americans, as well as the integration of component for reducing sedentary behavior into the app are strongpoints of this application. Moreover, the research environment, training and mentorship plans and institutional commitment to the applicant are excellent. Further, as an early stage investigator, the applicant has been productive. This application has merit but there some concerns that relate to elements of the research plan that reduce enthusiasm.

1. CANDIDATE:

Strengths

- Evidence of productivity in research related to smoking cessation in Black Americans.
- Focus on culturally tailored smart phone interventions.
- Progress in career track as evidenced by appointment to a faculty position.

Weaknesses

- None.

2. CAREER DEVELOPMENT PLAN/CAREER GOALS AND OBJECTIVES:

Strengths

- Well-designed plan for training and mentorship.
- Structured coursework to facilitate greater expertise in area of research focus.
- Career goals well thought out and in line with training and mentoring plan.

Weaknesses

- None.

3. RESEARCH PLAN:

Strengths

- Inclusion of an app component to reduce sedentary behavior.
- Tailoring quit smoking content to African Americans.
- Use of pilot test participants to review changes to the HealthyCells app.

Weaknesses

- Unclear why sedentary behavior is not an inclusion criterion.
- No sample size justification given for the use of 30 participants. Feasibility studies also require sample size justification.
- The data analysis plan requires more clarity.

4. MENTOR(S), CO-MENTOR(S), CONSULTANT(S), COLLABORATOR(S):

Strengths

- The application proposes a strong mentoring team with requisite expertise in areas that will contribute to applicant’s career development and the success of the proposed research.
- Mentors express strong support in writing.

Weaknesses

- None.

5. ENVIRONMENT AND INSTITUTIONAL COMMITMENT TO THE CANDIDATE:

Strengths

- Excellent environment with access to resources to facilitate the success of the proposed research.
- Commitment of the institution to the candidate’s progress and success is provided in writing.

Weaknesses

- None.

Study Timeline for Clinical Trials (Specific to applications designated clinical trial on the electronic cover sheet)

Strengths

- Timeline is appropriate for accomplishing the study aims.

Weaknesses

- None.

Protections for Human Subjects

Acceptable Risks and Adequate Protections

- Detailed description of protections. There is acknowledgement of the potential for breach of confidentiality but does not say how such a breech will be handled.

Data and Safety Monitoring Plan (Applicable for Clinical Trials Only):

Acceptable

- The plan provides an acceptable outline of how potential adverse outcomes and experiences will be addressed.

Inclusion Plans

- Sex/Gender: Distribution justified scientifically
- Race/Ethnicity: Distribution justified scientifically
- For NIH-Defined Phase III trials, Plans for valid design and analysis: Not applicable
- Inclusion/Exclusion Based on Age: Distribution justified scientifically
- The justifications for inclusion of participants by gender, ethnicity and age are in keeping with the public health goals of the research and are acceptable

Vertebrate Animals

Not Applicable (No Vertebrate Animals)

Biohazards

Not Applicable (No Biohazards)

Resubmission

- This is a resubmission by the applicant. The applicant has provided a detailed response to the critiques of previous reviewers that addresses each of the weakness that were noted.

Training in the Responsible Conduct of Research

Acceptable

Comments on Format (Required):

- The applicant has received institutional training in responsible conduct of research offered through the CITI initiative.

Comments on Subject Matter (Required):

- No specific information is provided on the content of training received through the institutional CITI program. Detailed information is provided on proposed supplemental training which is acceptable.

Comments on Faculty Participation (Required; not applicable for mid- and senior-career awards):

- Description of faculty/mentor participation in the applicant's training is acceptable.

Comments on Duration (Required):

- No information is provided on the duration of the formal institutional responsible conduct of research training. Proposed efforts to seek supplemental training in research throughout the period of the award is acceptable.

Comments on Frequency (Required):

- No information is provided on the institutional requirement on certification in responsible conduct of research. The proposed attendance of weekly workshops on the topic is acceptable.

Select Agents

Not Applicable (No Select Agents)

Resource Sharing Plans: No comment noted.

Authentication of Key Biological and/or Chemical Resources

Not Applicable (No Relevant Resources)

Budget and Period of Support

Recommended budget modifications or possible overlap identified:

- The project coordinator's effort at 10 percent seems low. The level of effort/support for this individual may need to be increased.

CRITIQUE 2

Candidate: 2
Career Development Plan/Career Goals /Plan to Provide Mentoring: 3
Research Plan: 5
Mentor(s), Co-Mentor(s), Consultant(s), Collaborator(s): 3
Environment Commitment to the Candidate: 1

OVERALL IMPACT: This K01 submission is proposing to develop this applicant's potential to become an independent investigator focused on reducing health disparities among African Americans by using smartphone / mHealth interventions that address smoking and other health risk to promote engagement in healthy behavior. The candidate has a strong early career background and has been highly productive in scholarship. The mentor team is solid and includes a primary mentor and five co-mentors who have substantial expertise that cover content and training areas. They also have mentorship experience. The training plan is well-outlined and consists of coursework, seminars, short courses, conferences, directed readings with mentors, practical experience from proposed K01 research study, and use of an individual development plan. The research plan is to adapt existing evidence-based smartphone interventions to be culturally appropriate for African American smokers to support smoking cessation. Content will be adapted using a literature review and pilot testing. A single-group design pre/post feasibility intervention study will then be conducted to examine pre/post change in point prevalence smoking abstinence and sedentary time 8 weeks later. Although the training plan includes directed readings with mentors, there is a lack of description of apprentice/lab experience(s) with mentor(s) as part of training plan that could enhance the candidate's practical and real-world training experiences in area of focus. There are a number of moderate concerns about the research plan. Specifically, methods to conduct and analyze findings from the literature review to inform content is not described, concern whether literature review is the best approach to develop content, steps to corroborate qualitative data analysis is not described, measures for survey questions not described and other questions on acceptability/satisfaction missing, rationale for conducting single-group design not well described and how these findings will inform next steps.

1. CANDIDATE:

Strengths

- Strong training in psychology and public health at the University of Memphis, including doctoral training in social and behavioral health sciences; postdoctoral training at the at the Oklahoma University Health Sciences Center (OUHSC) and currently tenure-track position at the OUHSC Department of Family and Preventive Medicine.
- Interest in using mobile health (mHealth) and use of smartphone interventions with specific goal of understanding and intervening on the behavioral determinants underlying health disparities (e.g., smoking-related illnesses and sedentary lifestyle) among African Americans.
- Focused on addressing health disparities among African Americans.
- Demonstrated record of publication.

Weaknesses

- None.

2. CAREER DEVELOPMENT PLAN/CAREER GOALS AND OBJECTIVES;

Strengths

- Use of an individual development plan and regular review of plan with mentors.
- Training plan consists of coursework, seminars, short courses, conferences, directed readings with mentors, practical experience from proposed K01 research study.
- Described training plan consists of strong activities.

Weaknesses

- Lack of description of apprentice/lab experience(s) with mentor(s) as part of training plan.

3. RESEARCH PLAN:

Strengths

- Significance of research area well-articulated; a major gap exists in interventions that engage African Americans to stop smoking and that promote sedentary behaviors.
- Innovative approach including use of automated incentives-based smartphone intervention for smoking cessation, intervention focused on behavior reduction.
- Creating culturally tailored treatment content for African American smokers that is integrated into a smartphone application for smoking cessation (i.e., HealthyCells) of two pre-existing evidence-based smartphone interventions, PrevailGO and SMARTpath; and evaluating the single-group design pre/post feasibility of HealthyCells at addressing smoking and sedentary behavior among African Americans.

Weaknesses

- Rationale for choice of mHealth approach over other approaches.
- Aim 1 methods to conduct literature review to develop culturally relevant content and how results from this review will be analyzed / inform content not well described; rationale for conducting a literature review over other potential methods (e.g., consensus building methodology with experts in the field and formative feedback by end users) not considered. Need to develop content and tailor based on a participant’s sex, age, or other relevant characteristics not well discussed.
- Aim 1 qualitative analysis plans lacks description of steps to corroborate qualitative data analysis findings, including verification of coding/themes by other investigator(s). Use of N-VIVO for qualitative analysis may not be appropriate to analyze type of data collected.
- Aim 1 lacks justification for sample size approach for qualitative study.
- Lack of scientific rationale for not assessing preliminary efficacy as part of Aim 2 and power calculations.
- Aim 2 survey questionnaire measures not described.
- Acceptability/satisfaction measures not included.
- Aim 2 discussion about potential confounders (e.g. sex) mentioned, but recruitment approach does not account for this and Aim 2 overall lacks justification for sample size approach for feasibility study.

4. MENTOR(S), CO-MENTOR(S), CONSULTANT(S), COLLABORATOR(S):

Strengths

- Solid team, including primary mentor, with extensive funding record and expertise related to conducting contingency management interventions for smoking cessation with socioeconomically disadvantaged populations, mHealth interventions, data collection procedures to monitor the smoking patterns of current menthol smokers, biostatistics, qualitative research, and approaches to promote physical activity and sedentary behavior reduction.

Weaknesses

- Track record for mentor/co-mentor mentees’ becoming independent investigators not well described.

5. ENVIRONMENT AND INSTITUTIONAL COMMITMENT TO THE CANDIDATE:

Strengths

- Excellent resources and institutional commitment.

Weaknesses

- None.

Study Timeline for Clinical Trials (Specific to applications designated clinical trial on the electronic cover sheet)

Strengths

- Designed as a single-group design feasibility study not designed nor powered to evaluate the efficacy of the intervention for smoking cessation and sedentary behavior reduction, inclusive of receipt of nicotine replacement therapy (NRT).

Weaknesses

- Three years is planned to implement and evaluate a feasibility trial of 30 participants.

Protections for Human Subjects

Acceptable Risks and Adequate Protections

- Acceptable

Data and Safety Monitoring Plan (Applicable for Clinical Trials Only):

Acceptable

Inclusion Plans

- Sex/Gender: Distribution justified scientifically
- Race/Ethnicity: Distribution justified scientifically
- For NIH-Defined Phase III trials, Plans for valid design and analysis: Not applicable
- Inclusion/Exclusion Based on Age: Distribution justified scientifically
    - Acceptable

Vertebrate Animals

Not Applicable (No Vertebrate Animals)

Biohazards

Not Applicable (No Biohazards)

Resubmission

- Responsive to prior comments. Major revisions were made based on prior review comments that the research plan was difficult to follow, how the qualitative study informed the clinical trial, and related to the experimental design of the intervention. Revisions were not made to provide financial rewards for sedentary behavior reduction. A number of additional minor revisions were also made. All of the revisions appear appropriate and responsive to reviewers' comments.

Although certain aspects of the application are improved, especially related to the candidate's career development plan, there are other issues as described above

Training in the Responsible Conduct of Research

Acceptable

Comments on Format (Required):

- Acceptable

Comments on Subject Matter (Required):

- Acceptable

Comments on Faculty Participation (Required; not applicable for mid- and senior-career awards):

- Faculty participation not well described

Comments on Duration (Required):

- Acceptable

Comments on Frequency (Required):

- Acceptable

Select Agents

Not Applicable (No Select Agents)

Resource Sharing Plans

Acceptable

Authentication of Key Biological and/or Chemical Resources

Not Applicable (No Relevant Resources)

Budget and Period of Support

Recommended budget modifications or possible overlap identified:

- Completion of proposed two study aims does not require a five-year time frame.
- Total maximum remuneration amount per participant is not clearly delineated; however, the budget lists upwards of $530 per participant or $397.50 at 75 percent reduction, which is rather generous. A concern is raised about the use of this approach.

CRITIQUE 3

Candidate: 2
Career Development Plan/Career Goals /Plan to Provide Mentoring: 1
Research Plan: 4
Mentor(s), Co-Mentor(s), Consultant(s), Collaborator(s): 1
Environment Commitment to the Candidate: 2

OVERALL IMPACT: The primary objective of this application is to build and evaluate a culturally tailored smartphone application for smoking cessation with adjunctive treatment for sedentary behavior. There are five training goals: to develop mastery in (1) culturally tailored intervention development; (2) mHealth methodology; (3) designing and evaluating interventions for sedentary behavior reduction; (4) intensive longitudinal data analysis; and (5) clinical trial design and evaluation. The research plan has been reduced to two aims. Research Aim 1 involves (a) configuring the smartphone intervention, (b) creating culturally tailored treatment content, and (c) conducting a formative study to assist with refining and finalizing the intervention for the clinical trial. Research Aim 2 involves evaluating the feasibility and deriving preliminary estimates of the effect of the HealthyCells app on smoking and sedentary behavior among African Americans. (2) The revision includes numerous Tables and Figures to summarize the information presented. This application has many strengths that were also apparent in the first application. The candidate is strong and well-positioned to benefit from this award. His letters of recommendation and mentor statements underscore his potential and how their roles will benefit the training and research plans. The training plan has a good combination of course work, conferences, manuscript writing, and individual mentoring across goals. There is also a strategy to evaluate the candidate's productivity. The research plan also appears to be much improved from the first submission with much greater specificity. The combination of reducing sedentary activity and a smartphone-based contingency management is innovative and sensible. There is relative clarity on how the research processes and qualitative studies will inform the development of the intervention. However, the research plan continues to have some weaknesses including questions about how the participants will manage all of the requirements and some lack of clarity regarding how well the qualitative analysis plan matches getting feedback on the ap. It is unclear whether there will be sufficient numbers of Black patients to meet recruitment goals. Finally, the absence of any kind of control is problematic.

1. CANDIDATE:

Strengths

- Reference letters (e.g., Drs. Ward, Ahluwalia, and Stern) underscore the candidate's qualifications, commitment, and potential.
- Good number of first authored papers in related area with 22 publications all together.
- Received a $7500 trainee research grant from the Stephenson Cancer Center, competitive selection to attend the Russell Sage Foundation Summer Institute on Biological Approaches in the Social Sciences (June 2019) and the National Institute on Minority Health and Health Disparities Research Institute (August 2019).
- Consistent record of research interest and productivity.

Weaknesses

- No comment noted.

2. CAREER DEVELOPMENT PLAN/CAREER GOALS AND OBJECTIVES:

Strengths

- High likelihood that the plan will contribute substantially to the scientific development of the candidate and lead to scientific independence.
- prior training and research experience appropriate for this award.
- The content, scope, phasing, and duration of the career development plan are appropriate when considered in the context of prior training/research experience and the stated training and research objectives for achieving research independence. Good combination of course work, conferences, manuscript writing, and individual mentoring across goals.

- There are adequate plans for monitoring and evaluating the candidate's research and career development progress—will have indicators and Dr. Kendzor will organize a quarterly benchmark meeting.
- High likelihood that experience with clinical trials will contribute to the applicant's research career development.
- Individual development plan (IDP) for each year of the award.

Weaknesses

- No comment noted.

3. RESEARCH PLAN:

Strengths

- No studies have attempted to promote sedentary behavior reduction as a cessation strategy.
- Prior research that serves as the key support for the proposed project is rigorous. Specifically, research team has developed numerous evidence-based smartphone interventions to address a range of modifiable health risk factors among vulnerable populations, which are capable of being culturally tailored for African Americans. Candidate assisted with the development and evaluation of a smartphone-based contingency management intervention that provided financial rewards for smoking abstinence after remote verification of smoking status using low-cost portable carbon monoxide monitors and facial recognition software (i.e., PrevailGO).
- Team also developed a smartphone-based sedentary behavior reduction intervention that uses wrist-worn activity monitors in combination with smartphone technology to monitor activity in real-time and deliver activity prompts when individuals are engaged in prolonged bouts of sedentary behavior (i.e., SMARTpath). SMARTpath also provides graphical feedback about sedentary time over the past day, week, and month. Integrating and tailoring these evidence-based smartphone interventions for African Americans may equip them with the tools, skills, and information needed to remain abstinent from smoking.
- The research plan is relevant to the candidate's research career objectives.
- Innovations include automated incentives based smartphone intervention for smoking cessation, the addition of a sedentary behavior reduction intervention is novel and optimizes the potential for improved health, the proposed smartphone intervention reduces barriers to treatment and maximizes convenience for participants, and this will be the first smoking cessation intervention to use sedentary behavior reduction as a strategy to increase cessation rates. Despite a high prevalence of smartphone ownership among African Americans,150 this smartphone intervention will be the first smoking cessation app that is tailored specifically for African American smokers.
- Creation of prototype by reviewing literature and doing demo in 15 participants.

Weaknesses

- Use of literature review to define elements of adaptation seems limited. There may be advantages to getting information and preferences from potential end users and other experts.
- It is not clear to me that working with the ap for an hour can give a person the chance to see how it can really be used. (May be standard) There did not seem to be any consideration given to sampling.
- The number of Black participants at the Tobacco Treatment Research Program (TTRP) is estimated at about 105 per year, making it hard to achieve recruitment goal of eight to ten per month for Aim 1. It is difficult to know if they will be able to recruit four to six participants per month for the open trial, especially given the inclusion criteria.

- It is unclear how participants will manage the requirements of intervention and how they will be monitored.
- It would have been helpful to include a diagram of the conceptual framework.
- It is unclear if there are any contraindications for NRT.
- It is not clear that Nvivo is the best method for analyzing data to uncover what is needed for the intervention.
- Absence of control group or condition is problematic.

4. MENTOR(S), CO-MENTOR(S), CONSULTANT(S), COLLABORATOR(S):

Strengths

- An excellent mentoring team with relevant expertise and research record.
- Dr. Darla E. Kendzor will provide expertise in the areas of tobacco cessation and sedentary behavior interventions, clinical trials, contingency management, health disparities, and mobile health. Has excellent research record.
- Dr. Michael S. Businelle will help in the development of the HealthyCells app using the InsightTM mHealth platform (Ain 1 and Training Goal B), and provide guidance on managing and evaluating clinical trials (Training Goal E).
- Dr. Summer Gale Frank-Pearce will provide biostatistical expertise and works closely with the overall team.
- Dr. Marshall Cheney will provide training in qualitative methods.
- Dr. Lorna Haughton-McNeil, the Professor and Chair, Department of Health Disparities Research, will help on how to develop culturally specific content for increasing physical activity and reduction of sedentary behavior.
- Dr. Amy Cohn will help develop culturally tailored content for smoking cessation intervention, with a focus on the role of menthol use in smoking and cessation as well as on using smartphones for ecological momentary assessment and intensive longitudinal data, and provide mentorship on implementing clinical trials related to cigarette use.
- Adequate expertise on conducting clinical trial.

Weaknesses

- No comment noted.

5. ENVIRONMENT AND INSTITUTIONAL COMMITMENT TO THE CANDIDATE:

Strengths

- The Oklahoma University Health Sciences Center (OUHSC) and Oklahoma Tobacco Research Center (OTRC) seem excellent for the candidate and his study.
- Candidate has been offered a tenure-track faculty position at the Department of Family and Preventive Medicine beginning on August 1st, 2020.

Weaknesses

- No comment noted.

Study Timeline for Clinical Trials (Specific to applications designated clinical trial on the electronic cover sheet)

Strengths

- Good use of resources available in Tobacco Treatment Research Program.
- Overall timeline is reasonable.

Weaknesses

- Concern is raised about the lack of any recruitment/retention strategies as may have trouble recruiting for both segments. It would be good to have back-up and monitoring plans.

Protections for Human Subjects

Acceptable Risks and Adequate Protections

- Risks include confidentiality, NRT, and withdrawal symptoms.

Data and Safety Monitoring Plan (Applicable for Clinical Trials Only):

Acceptable

- Reasonable

Inclusion Plans

- Sex/Gender: Distribution justified scientifically
- Race/Ethnicity: Distribution justified scientifically
- For NIH-Defined Phase III trials, Plans for valid design and analysis: Not applicable
- Inclusion/Exclusion Based on Age: Distribution justified scientifically
  - Exclusion of children and inclusion of only African Americans are justified.

Vertebrate Animals

Not Applicable (No Vertebrate Animals)

Biohazards

Not Applicable (No Biohazards)

Resubmission

- The research plan has been reduced to two aims. Research Aim 1 involves (a) configuring the smartphone intervention, (b) creating culturally tailored treatment content, and (c) conducting a formative study to assist with refining and finalizing the intervention for the clinical trial. Research Aim 2 involves evaluating the feasibility and deriving preliminary estimates of the effect of the HealthyCells app on smoking and sedentary behavior among African Americans. The revision includes numerous tables and figures to summarize the information presented.

Training in the Responsible Conduct of Research

Acceptable

Comments on Format (Required):

- Use of multiple formats.

Comments on Subject Matter (Required):

- Reasonable subject matter.

Comments on Faculty Participation (Required; not applicable for mid- and senior-career awards):

- Faculty are named, but more detail could be provided on what faculty will do (e.g., in relation to skill development, learning goals, research progress).

Comments on Duration (Required):

- This aspect is less clear.

Comments on Frequency (Required):

- Provided for different components.

Select Agents

Not Applicable (No Select Agents)

Resource Sharing Plans

Not Applicable (No Relevant Resources)

Authentication of Key Biological and/or Chemical Resources

Not Applicable (No Relevant Resources)

Budget and Period of Support

Recommend as Requested

THE FOLLOWING SECTIONS WERE PREPARED BY THE SCIENTIFIC REVIEW OFFICER TO SUMMARIZE THE OUTCOME OF DISCUSSIONS OF THE REVIEW COMMITTEE, OR REVIEWERS' WRITTEN CRITIQUES, ON THE FOLLOWING ISSUES:

STUDY TIMELINE FOR CLINICAL TRIALS: ACCEPTABLE

PROTECTION OF HUMAN SUBJECTS: ACCEPTABLE
The section is considered acceptable. Proposed participant payments are quite high, which may be perceived as coercive and could raise a human subjects' concern; however, it is assumed that such issues are addressed via IRB review of TRC protocols.

INCLUSION OF WOMEN PLAN: ACCEPTABLE

INCLUSION OF MINORITIES PLAN: ACCEPTABLE

INCLUSION ACROSS THE LIFESPAN PLAN: ACCEPTABLE

VERTEBRATE ANIMALS: NOT APPLICABLE

TRAINING IN THE RESPONSIBLE CONDUCT OF RESEARCH: ACCEPTABLE
The section is considered acceptable; but more detail and clarity are expected for each of the five elements.

COMMITTEE BUDGET RECOMMENDATIONS: The budget was recommended as requested.
The panel approved the budget as recommended. However, the following reviewer comments are noted.

- Study aims were reduced from three to two; it is unclear that completion of two proposed study aims will require the five-year time frame.
- Total maximum remuneration amount per participant is not clearly delineated; however, the budget lists upwards of $530 per participant or $397.50 at 75 percent reduction, which is rather generous.

Footnotes for 1 K01 MD015295-01A1; PI Name: Alexander, Adam C

NIH has modified its policy regarding the receipt of resubmissions (amended applications).See Guide Notice NOT-OD-18-197 at https://grants.nih.gov/grants/guide/notice-files/NOT-OD-18-197.html. The impact/priority score is calculated after discussion of an application by averaging the overall scores (1-9) given by all voting reviewers on the committee and multiplying by 10. The criterion scores are submitted prior to the meeting by the individual reviewers assigned to an application, and are not discussed specifically at the review meeting or calculated into the overall impact score. Some applications also receive a percentile ranking. For details on the review process, see http://grants.nih.gov/grants/peer_review_process.htm#scoring.

MEETING ROSTER

National Institute on Minority Health and Health Disparities Special Emphasis Panel
NATIONAL INSTITUTE ON MINORITY HEALTH AND HEALTH DISPARITIES
NIMHD K01 Career Development Award Peer Review

ZMD1 DRI (J1)
11/09/2020 - 11/10/2020

Notice of NIH Policy to All Applicants: Meeting rosters are provided for information purposes only. Applicant investigators and institutional officials must not communicate directly with study section members about an application before or after the review. Failure to observe this policy will create a serious breach of integrity in the peer review process, and may lead to actions outlined in NOT-OD-14-073 at https://grants.nih.gov/grants/guide/notice-files/NOT-OD-14-073.html and NOT-OD-15-106 at https://grants.nih.gov/grants/guide/notice-files/NOT-OD-15-106.html, including removal of the application from immediate review.

CHAIRPERSON(S)

DIXON, LISA B., MD
EDNA L. EDISON PROFESSOR
DEPARTMENT OF PSYCHIATRY
COLUMBIA UNIVERSITY
DIRECTOR, CENTER FOR PRACTICE INNOVATIONS
NEW YORK STATE PSYCHIATRIC INSTITUTE
NEW YORK, NY 10032

MEMBERS

BOUTJDIR, MOHAMED, PHD
DIRECTOR AND PROFESSOR
DEPARTMENT OF MEDICINE
CARDIOVASCULAR RESEARCH PROGRAM
SUNY DOWNSTATE MEDICAL CENTER
NEW YORK UNIVERSITY SCHOOL OF MEDICINE
NEW YORK, NY 11203

COCHRAN, SUSAN D, PHD
PROFESSOR
DEPARTMENT OF EPIDEMIOLOGY
FIELDING SCHOOL OF PUBLIC HEALTH
UNIVERSITY OF CALIFORNIA, LOS ANGELES
LOS ANGELES, CA 90095

EVERETT, BETHANY, PHD
ASSOCIATE PROFESSOR
DEPARTMENT OF SOCIOLOGY
COLLEGE OF SOCIAL & BEHAVIORAL SCIENCES
THE UNIVERSITY OF UTAH
SALT LAKE CITY, UT 84108

HEADEN, IRENE
ASSISTANT PROFESSOR OF BLACK HEALTH
DEPARTMENT OF COMMUNITY HEALTH AND PREVENTION
DORNSIFE SCHOOL OF PUBLIC HEALTH
DREXEL UNIVERSITY
PHILADELPHIA, PA 19104

HUEBNER, DAVID M, PHD
ASSOCIATE PROFESSOR
DEPARTMENT OF PREVENTION AND COMMUNITY HEALTH
MILKEN INSTITUTE
SCHOOL OF PUBLIC HEALTH
THE GEORGE WASHINGTON UNIVERSITY
WASHINGTON, DC 20052

JENNINGS, JULIA, PHD
ASSOCIATE PROFESSOR
DEPARTMENT OF ANTHROPOLOGY
STATE UNIVERSITY OF NEW YORK AT ALBANY
ALBANY, NY 12202

MARCELL, ARIK V, MD
ASSOCIATE PROFESSOR
DIVISION OF ADOLESCENT/YOUNG ADULT MEDICINE
DEPARTMENTS OF PEDIATRICS AND
POPULATION, FAMILY, AND REPRODUCTIVE HEALTH
JOHNS HOPKINS UNIVERSITY
BALTIMORE, MD 21287

MCHALE, JAMES P, PHD
ASSOCIATE PROFESSOR AND DIRECTOR
DEPARTMENT OF PSYCHOLOGY
FAMILY STUDY CENTER
UNIVERSITY OF SOUTH FLORIDA
ST. PETERSBURG, FL 33701

O'BRIEN, SARAH H, MD
ASSOCIATE PROFESSOR
OF PEDIATRICS
DIVISION OF HEMATOLOGY/ONCOLOGY/BMT
THE RESEARCH INSTITUTE AT
NATIONWIDE CHILDREN'S HOSPITAL
COLUMBUS, OH 43205

OSER, CARRIE BETH, PHD
INTERIM ASSOCIATE DEAN FOR RESEARCH; PROFESSOR AND ASSOCIATE CHAIR
DEPARTMENT OF SOCIOLOGY
CENTER ON DRUG AND ALCOHOL RESEARCH
COLLEGE OF ARTS AND SCIENCES
UNIVERSITY OF KENTUCKY
LEXINGTON , KY 40506

PALANIAPPAN, LATHA P, MD
PROFESSOR
DEPARTMENT OF MEDICINE
SCHOOL OF MEDICINE
STANFORD UNIVERSITY
PALO ALTO, CA 94301

RASMUSSEN, ANDREW EDWARD, PHD
ASSOCIATE PROFESSOR
DEPARTMENT OF PSYCHOLOGY
FORDHAM UNIVERSITY
BRONX, NY 10458

SHREFFLER, KARINA M., PHD
PROFESSOR
TULSA ACADEMIC PROGRAMS COORDINATOR
DEPARTMENT OF HUMAN DEVELOPMENT AND FAMILY SCIENCE
COLLEGE OF HUMAN SCIENCES
OKLAHOMA STATE UNIVERSITY-TULSA
TULSA, OK 74106

YELLOWLEES, PETER M, MD, MBBS
ALLAN STOUDEMIRE ENDOWED PROFESSOR
DEPARTMENT OF PSYCHIATRY AND BEHAVIORAL SCIENCES
CHIEF WELLNESS OFFICER, UCD HEALTH
UNIVERSITY OF CALIFORNIA DAVIS
SACRAMENTO, CA 95817

MAIL REVIEWER(S)

TULL, EUGENE S, DRPH
EXECUTIVE DIRECTOR AND SENIOR RESEARCH FELLOW
INTERAMERICAN CENTER FOR PUBLIC HEALTH IMPROVEMENT
CHRISTIANSTED, VI
ADJUNCT ASSOCIATE PROFESSOR OF AFRICANA STUDIES
UNIVERSITY OF PITTSBURGH
PITTSBURGH, PA 15261

SCIENTIFIC REVIEW OFFICER

ISMOND, DEBORAH, PHD
SCIENTIFIC REVIEW OFFICER
SCIENTIFIC REVIEW BRANCH
OFFICE OF EXTRAMURAL RESEARCH ADMINISTRATION
NATIONAL INSTITUTE ON MINORITY HEALTH AND HEALTH DISPARITIES, NATIONAL INSTITUTES OF HEALTH
BETHESDA, MD 20892

EXTRAMURAL SUPPORT ASSISTANT

DAVIS, CRYSTAL
SUPPORT ASSISTANT
SCIENTIFIC REVIEW BRANCH
OFFICE OF EXTRAMURAL RESEARCH ADMINISTRATION
NATIONAL INSTITUTE ON MINORITY HEALTH AND HEALTH DISPARITIES, NATIONAL INSTITUTES OF HEALTH
BETHESDA, MD 20892

Consultants are required to absent themselves from the room during the review of any application if their presence would constitute or appear to constitute a conflict of interest.
